# Oral commercial Chinese polyherbal preparations combined with conventional biomedicine for pulmonary tuberculosis: network meta-analysis

**DOI:** 10.3389/fphar.2025.1588586

**Published:** 2025-10-28

**Authors:** Jiankang Jiang, Siqi Zhang, Hui Wang, Lu Zhang, Zegeng Li, Jiabing Tong, Fan Wu

**Affiliations:** ^1^ School of Traditional Chinese Medicine, Anhui University of Chinese Medicine, Hefei, China; ^2^ Anhui Xinhua University, Hefei, China; ^3^ Anhui Provincial Key Laboratory of Applied Transformation for Prevention and Treatment of Major Pulmonary Diseases with Traditional Chinese Medicine, Hefei, China; ^4^ The First Affiliated Hospital of Anhui University of Chinese Medicine, Hefei, China; ^5^ Institute of Prevention and Treatment of Respiratory Diseases, Anhui Provincial Academy of Chinese Medical Sciences, Hefei, China

**Keywords:** pulmonary tuberculosis, oral commercial Chinese polyherbal, biomedicine, combination therapy, network meta-analysis, randomized controlled trials

## Abstract

**Background:**

Despite therapeutic interventions, tuberculosis (TB) remains a persistent challenge. Combination therapy integrating Traditional Chinese Medicine (TCM) with conventional anti-TB medications demonstrates therapeutic benefits, necessitating a meta-analysis evaluating the adjunctive use of oral commercial Chinese polyherbal preparation (CCPP).

**Objectives:**

This study aims to evaluate the efficacy and safety of different oral TCMs combined with biomedicine for tuberculosis treatment, utilizing network meta-analysis techniques.

**Methods:**

A computer-based search was conducted across various databases including the China National Knowledge Infrastructure (CNKI), Wanfang Database, VIP Database, SinoMed, PubMed, EMbase, Web of Science, and the Cochrane Library, covering records from database inception to 22 January 2025. Data analysis utilized Stata 18.0, R software (version 4.4.1), and Review Manager 5.4.

**Results:**

A total of 100 randomized controlled trials (RCTs) were included, with a total sample size of 12,747 participants (6,639 in the experimental group and 6,108 in the control group), evaluating 12 distinct interventions. Network meta-analysis revealed the following optimal combinations: FeiJieHe Pill combined with standard biomedicine demonstrated superior efficacy for clinical response rate improvement (OR = 8.43, 95% CI [1.79, 39.69]). KangLao Pill combined with standard biomedicine was the most effective for negative conversion rate (OR = 11.55, 95% CI [3.04, 43.93]). Bu Jin Tablet combined with standard biomedicine demonstrated superior efficacy for lesion absorption rate (OR = 7.46, 95% CI [3.32, 16.75]). FeiJieHe Pill combined with standard biomedicine was the most effective for cavity absorption (OR = 5.11, 95% CI [2.04, 12.85]). JieHe Pill combined with conventional biomedicine yielded the greatest improvement in both CD3^+^ T lymphocyte response (OR = 5.6, 95% CI [3.4, 7.8]) and CD4^+^ T lymphocyte response (OR = 5.1, 95% CI [2.9, 7.3]).

**Conclusion:**

Combination therapy utilizing oral CCPP alongside conventional biomedicine has a significant enhanced efficacy relative to conventional biomedicine monotherapy across multiple tuberculosis treatment metrics, including clinical efficacy rate, negative conversion rate, lesion absorption rate, cavity absorption rate, and improvement rates of CD3^+^ and CD4^+^ T lymphocyte levels.

**Systematic Review Registration:**

https://www.crd.york.ac.uk/prospero/display_record.php?ID=CRD42024589122, identifier CRD42024589122.

## 1 Introduction

Tuberculosis (TB), an airborne infectious disease caused by *Mycobacterium tuberculosis*, persists as a major global health threat in 21st century and remains one of the key infectious diseases controlled in China. According to the World Health Organization’s 2023 report (Wei and LiuYuhon, 2024), global TB incidence reached 10.6 million new cases in 2022, including 748,000 cases in China, ranking the third among high-burden nations. *Bacillus* Calmette-Guérin (BCG) vaccination programs and standardized modern pharmaceutical interventions render the TB epidemic both preventable and controllable. However, the emergence of drug-resistant strains, particularly multidrug-resistant tuberculosis, poses new challenge to TB control (Hui et al., 2020). Currently, biomedicine primarily treats TB with oral anti-TB medications, emphasizing early, combined, appropriate, regular, and full-course use. The conventional 2HRZE/4HR regimen comprises 2-month of therapy with Isoniazid, Rifampicin, Pyrazinamide, and Ethambutol followed by 4 months of Isoniazid and Rifampicin. While demonstrating clinical efficacy, long-term use of this regimen carries potential multi-organ toxicity risks (Shah et al., 2020; Prasad et al., 2019). Furthermore, treatment discontinuation secondary to adverse drug effects in newly diagnosed patients may predispose to develop drug-resistant TB development. A study by Zou Xia-li et al. (Xiali et al., 2017) identified a 2.38-fold increased risk of drug resistance development among TB patients experiencing anti-TB drug-related adverse reactions during initial treatment, with this the risk increased to 4.20-fold in individuals with prior treatment-associated adverse reactions. Correspondingly, Wei et al. (Wei et al., 2015) determined that anti-TB drug adverse reactions constitute the primary cause of treatment discontinuation in this patient population. Improving the efficacy of anti-TB drugs and reducing their side effects has become an urgent issue in modern TB management.

TB corresponds to the “pulmonary tuberculosis” classification within Traditional Chinese Medicine (TCM). Specialized texts such as Shí Yào Shén Shū (Ten Medicines Divine Book) of the Yuan Dynasty (Ge, 2019) documented early therapeutic approaches. The treatment principles of “tonifying the deficiency” and “killing parasites” established in Yīxué Zhèng Zhuàn (The Correct Transmission of Medicine) of the Ming Dynasty remain clinically relevant. TCM posits pulmonary tuberculosis as a chronic infectious disease caused by vital energy deficiency and infection of “consumption worms” that invade the lungs. Previous studies demonstrate that synergistic integration of TCM and biomedicine enhances the efficacy of anti-TB drugs, shortens treatment time, and mitigates side effects. Zhang Zhi-jie et al. (Zhijie, 2018) found that compared with simply using biomedicine, Qinghao Biejia Tang significantly improved the cure rate for drug-resistant TB. Similarly, Kang Guannan et al. (Guannan et al., 2023) observed higher cure rate, negative conversion rate, and cavity improvement rate following Kangfuxin Liquid combination therapy relative to anti-TB drugs alone, while also alleviating hemoptysis symptoms and reducing side effects.

Although multiple studies support enhanced treatment outcomes and reduced toxicity from adjunctive administration of oral traditional commercial Chinese polyherbal preparation (CCPP) alongside anti-TB biomedicine, the optimal therapeutic profile and comparative advantages of specific formulations remain undetermined. This study conducts a network meta-analysis comparing the efficacy of different oral CCPP as adjunctive therapy to anti-TB drugs, aiming to provide better treatment options for clinical practice.

## 2 Materials and methods

This study protocol received registration on the PROSPERO platform with the registration number CRD42024589122.

### 2.1 Literature search

To perform literature search, we have used multiple databases like China National Knowledge Infrastructure (CNKI) (https://oversea.cnki.net/index/), Wanfang (http://www.wanfangdata.com/), VIP Full Text Journals Database (https://www.fjlib.net/English/digitalresources/201804/t20180414_349923.htm), SinoMed (https://www.sinomed.com/), PubMed (https://pubmed.ncbi.nlm.nih.gov/), Embase (https://www.embase.com), Web of Science (https://www.webofscience.com), and Cochrane Library (https://www.cochranelibrary.com/). The databases were screen up to 22 January 2025, based on a robust literature screening and data extraction strategy. The languages considered were Chinese and English.

### 2.2 Literature screening and data extraction

Three researchers participated in the literature screening and data extraction, with one designated as the group leader. In the event of any disagreement during the screening process, the group leader was responsible for making the final decision. According to the diagnostic criteria for pulmonary tuberculosis (Chuanjun et al., 2023; Guofang and Xiwei, 2020; Radiology, 2018), a confirmed diagnosis required meeting at least one of the following conditions, along with mandatory fulfillment of condition C:

A. Persistent cough and sputum production lasting for 2 weeks or more, with or without hemoptysis, blood-streaked sputum, or systemic symptoms.
B. Radiographic findings suggestive of pulmonary tuberculosis.
C. Detection of *Mycobacterium tuberculosis* in sputum or other specimens, confirmed by smear microscopy, culture, or molecular biological methods.

There were no restrictions regarding gender, age, prior treatment history (initial treatment or retreatment), or drug resistance status.

The control group received a standard anti-tuberculosis monotherapy regimen or monotherapy combined with adjunctive treatments such as hepatoprotective or hemostatic agents to alleviate complications. The experimental group was administered a single oral Chinese patent medicine in addition to the control regimen. The total treatment duration was the same for both groups.

Studies were excluded if they met any of the following criteria: non-randomized controlled trials (non-RCTs), narrative reviews, systematic reviews, conference abstracts, clinical guidelines, studies with undefined treatment durations, animal or *in vitro* experiments, academic theses, or topics for which fewer than three relevant studies were available.

The outcome indicators collected included the following: A. Clinical efficacy rate; B. Sputum conversion rate; C. Lesion absorption rate; D. Cavity closure rate; E. Improvement rate of CD3^+^ T lymphocytes; F. Improvement rate of CD4^+^ T lymphocytes; G. Incidence of adverse reactions.

### 2.3 Literature quality assessment

Methodological quality evaluation of included RCTs utilized Cochrane Handbook standards (Higgins et al., 2023) assessing dimensions of randomization method, allocation concealment, blinding of participants and staff, blinding of outcome assessors, completeness of data, selective reporting of outcomes, and other biases. If there were any disagreements during the evaluation, consensus was reached through in-depth discussion with the group leader. Each evaluation item was classified as “low risk”, “unclear”, or “high risk”. All results were systematically documented within RevMan 5.4 software (Cochrane Collaboration, 2020), facilitating generation of corresponding risk of bias visualizations.

### 2.4 Statistical analysis

A network meta-analysis was conducted using the “netmeta” package (Rücker et al., 2024) in R (version 4.4.1) (R Core Team, 2024), and forest plots were generated.Binary outcomes employed odds ratio (OR) as the effect size measure, while continuous outcomes utilized standardized mean difference (MD), with reported 95% confidence intervals (CI). If the 95% CI for a binary variable does not include 1, or the 95% CI for a continuous variable does not include 0, statistical significance between groups was considered to be present. Evidence network were constructed using Stata 18.0 (StataCorp, 2023), featuring nodes proportional to intervention-specific sizes and interconnecting. To rank the effectiveness of different interventions, the Surface Under the Cumulative Ranking Curve (SUCRA) was used, with higher SUCRA values indicating a greater probability of being among the most effective treatments. No fixed SUCRA threshold was applied. This interpretation follows established methodological guidance in network meta-analysis (Salanti et al., 2011; Rücker and Schwarzer, 2015). Additionally, comparison-corrected funnel plots were drawn to examine publication bias in the included studies. Additional details are provided in the Supplementary Material (Supplementary Files 1–3).

## 3 Results

### 3.1 Literature search

**FIGURE 1:**
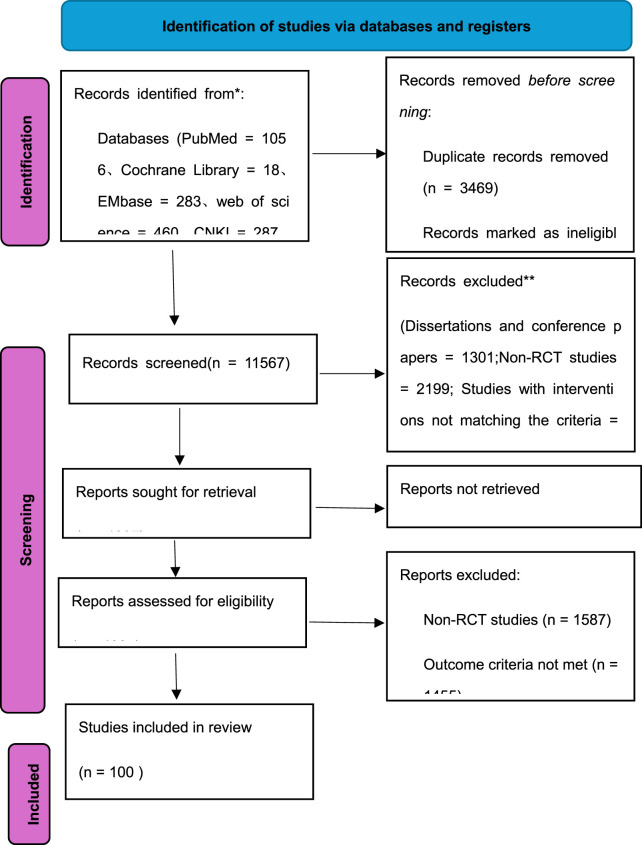
PRISMA flow diagram of the studies selection process.

A total of 15,036 articles were identified, with 100 articles meeting final inclusion criteria as exclusively two-arm RCTs. For multiple reports of the same study (such as clinical trial registration records and journal articles), the version with the most comprehensive or recent iteration was retained, and related studies were merged before data extraction. The literature screening flowchart is shown in Figure 1.

### 3.2 Characteristics of included literature

The 100 included articles (Jian, 2002; Yanpei and Shuyu, 2002; Ming, 2004; Wei et al., 2004; Donghua and Ganhong, 2005; Liuxing, 2005; Mengqiu et al., 2006; Lidong and Shuqing, 2007; Wulu et al., 2007; Xuewen, 2007; Yujuan, 2007; Ge et al., 2009; Kai, 2009; Shouyue, 2009; Xian, 2009; Xiuxia, 2009; Fuyuan and MeiMingxing, 2010; Guiqiu, 2010, Jun, 2010; Bing and Yuanai, 2011; Shiqi, 2011; Fengyan, 2012; Xinhua, 2012; Dan et al., 2013; Hualiang, 2013; Hunxi and Zhanqi, 2013; Lidong et al., 2013; Wen-ge et al., 2013; Xuhua et al., 2013; Zhongming, 2013; Long et al., 2014; Qianxin et al., 2014; Qing and Wengfang, 2014; Xiue et al., 2014; Jinghua et al., 2015; Min-shuang et al., 2015; Tianfeng, 2015; Yunxiao and Guosheng, 2015; Yuxia, 2015; Juan et al., 2016; Junguo et al., 2016; Shiming et al., 2016; Shuang, 2016; Shuhua and Fujian, 2016; Wei, 2016; Zhenru, 2016; Ji et al., 2017; Jun et al., 2017; Xing et al., 2017; Yongyi et al., 2017; Junjie, 2018; Lingwei, 2018; Liping, 2018; Xiaoni, 2018; Xiaoru et al., 2018; Di et al., 2019; Guizhen, 2019; Hai-xian et al., 2019; Junmei et al., 2019; Meng, 2019; Qingfeng, 2019; Zhibo et al., 2019; Bin, 2020; Guannan et al., 2020; Hui et al., 2020; Shuangli et al., 2020; Weiyong et al., 2020; Xian-hui and Shu-lin, 2020; Qiang, 2021; Qiong et al., 2021; Wei-wei, 2021; Chenyong et al., 2022; Jiafu and Lanhua, 2022; Juan, 2022; Qihuang, 2022; Jun et al., 2022; Xiaohua, 2022; Xuehu et al., 2022; Xueyun, 2022; Chang et al., 2022; Guannan et al., 2023; Chuanjun et al., 2023; Junguo, 2023; Qiaoe et al., 2023; Yawei et al., 2023; Yuxia, 2023; Peiqian et al., 2024; Xinlei and Rong, 2024; Yuelian, 2024) were all two-arm experiments, with a total sample size of 12,747 participants: 6,639 in the experimental group and 6,108 in the control group. These studies involved 12 different interventions:

• Bai Ling Capsule: seven studies (Yanpei and Shuyu, 2002; Donghua and Ganhong, 2005; Ge et al., 2009; Juan et al., 2016; Shuhua and Fujian, 2016; Hui et al., 2020; Shuangli et al., 2020)
• Bu Fei Huo Xue Capsule: five studies (Tianfeng, 2015; Xiaoru et al., 2018; Junmei et al., 2019; Chuanjun et al., 2023; Yawei et al., 2023)
• Bu Jin Tablet: four studies (Jian, 2002; Xian, 2009; Jun, 2010; Yunxiao and Guosheng, 2015)
• FeiJieHe Pill: five studies (Ming, 2004; Fengyan, 2012; Xinhua, 2012; Qing and Wengfang, 2014; Di et al., 2019)
• Fei Tai Capsule: seven studies (Donghua and Ganhong, 2005; Mengqiu et al., 2006; Hualiang, 2013; Junguo et al., 2016; Ji et al., 2017; Hai-xian et al., 2019; Xian-hui and Shu-lin, 2020)
• Jian Pi Run Fei Pill: seven studies (Wulu et al., 2007; Shouyue, 2009; Fuyuan and MeiMingxing, 2010; Bing and Yuanai, 2011; Hunxi and Zhanqi, 2013; Wei, 2016; Chenyong et al., 2022)
• Jie He Ling Tablet: four studies (Lidong and Shuqing, 2007; Zhongming, 2013; Shuang, 2016)
• Jie He Pill: 34 studies (Guiqiu, 2010; Shiqi, 2011; Dan et al., 2013; Lidong et al., 2013; Xuhua et al., 2013; Hunxi and Zhanqi, 2013; Qianxin et al., 2014; Min-shuang et al., 2015; Yuxia, 2015; Junguo et al., 2016; Juan et al., 2016; Zhenru, 2016; Xing et al., 2017; Yongyi et al., 2017; Lingwei, 2018; Liping, 2018; Guizhen, 2019; Meng, 2019; Bin, 2020; Chenyong et al., 2022; Jiafu and Lanhua, 2022; Juan, 2022; Jun et al., 2022; Chang et al., 2022; Junguo, 2023; Qiaoe et al., 2023; Yuxia, 2023; Peiqian et al., 2024; Xinlei and Rong, 2024; Yuelian, 2024)
• Kangfuxin Liquid: 10 studies (Wen-ge et al., 2013; Min-shuang et al., 2015; Xiaoru et al., 2018; Hai-xian et al., 2019; Zhibo et al., 2019; Guannan et al., 2020; Xiaohua, 2022; Xuehu et al., 2022; Xueyun, 2022; Guannan et al., 2023)
• KangLao Pill: five studies (Jinghua et al., 2015; Xiaoni, 2018; Qingfeng, 2019; Weiyong et al., 2020; Chenyong et al., 2022)
• Qi Jia Li Fei Capsule: six studies (Long et al., 2014; Xiue et al., 2014; Juan et al., 2016; Shiming et al., 2016; Jun et al., 2017; Junjie, 2018)
• Yi Fei Zhi Ke Capsule: six studies (Wei et al., 2004; Liuxing, 2005; Xuewen, 2007; Yujuan, 2007; Kai, 2009; Xiuxia, 2009)

The baseline characteristics of the two groups were comparable. The basic information of the included literature is shown in Table 1.

**TABLE 1:** Characteristics of the included randomized controlled trials.

Included in study	Sample size		Age (years)		Interventions		Treatment course	Outcome measures
	T	C	T	C	T	C		
Jian (2002)	31	28	—	—	BJ+Bio	Bio	9	①②
Yanpei and Shuyu (2002)	48	30	60–86	60–84	BL+Bio	Bio	3	①②⑤
Ming (2004)	50	50	14–60	16–59	FJH+Bio	Bio	6	①
Wei et al. (2004)	64	64	18–76	18–74	YFZK+Bio	Bio	6	①②
Donghua and Ganhong (2005)	62	60	26–67	25–66	BL+Bio	Bio	6	①②④
Liuxing (2005)	128	64	—	—	YFZK+Bio	Bio	6	①④
Pu et al. (2005)	38	15	—	—	FT+Bio	Bio	6	①②③
Mengqiu et al. (2006)	168	85	—	—	FT+Bio	Bio	8	①②③⑤⑥⑦
Xuewen (2007)	64	64	18–76	18–74	YFZK+Bio	Bio	6	①③④⑤
Lidong and Shuqing (2007)	75	75	—	—	JHL+Bio	Bio	6	①③⑤
Wulu et al. (2007)	241	100	—	—	JPRF+Bio	Bio	2	①②④⑤
Yujuan (2007)	67	65	—	—	YFZK+Bio	Bio	9	①②③⑤
Shuqin et al. (2008)	70	70	18–65	18–65	JHL+Bio	Bio	8	①③④⑤
Shouyue (2009)	168	85	18–72	18–72	JPRF+Bio	Bio	8	①③④⑤
Kai (2009)	53	52	—	—	YFZK+Bio	Bio	6	①②
Xian (2009)	30	30	19–65	25–68	BJ+Bio	Bio	3	①④
Xiuxia (2009)	132	130	21–68	20–70	YFZK+Bio	Bio	21	①②⑤
Ge et al. (2009)	123	121	16–67	18–66	BL+Bio	Bio	6	①②④⑤
Fuyuan and Meimingxing (2010)	90	90	X70	X70	JPRF+Bio	Bio	6	①②⑤
Guiqiu (2010)	36	38	—	—	JH+Bio	Bio	3	①②③⑤
Jun (2010)	26	22	—	—	BJ+Bio	Bio	6	①②③
Shiqi (2011)	30	26	60–87	61–88	JH+Bio	Bio	3	①
Bing and Yuanai (2011)	100	100	25–71	23–70	JPRF+Bio	Bio	8	①②③⑤
Xinhua (2012)	50	50	—	—	FJH+Bio	Bio	6	①②③④
Fengyan (2012)	32	32	—	—	FJH+Bio	Bio	12	①②⑤
Lidong et al. (2013)	50	50	—	—	JH+Bio	Bio	3	①②④
Wen-ge et al. (2013)	63	57	24–57	27–56	KF+Bio	Bio	6	①③⑤
Dan et al. (2013)	39	38	—	—	JH+Bio	Bio	6	①③④⑤
Hunxi and Zhanqi (2013)	100	90	11–75	19–68	JPRF+Bio	Bio	6	①⑤
Zhongming (2013)	75	75	—	—	JHL+Bio	Bio	6	①⑤
Hualiang (2013)	60	57	—	—	FT+Bio	Bio	6	①②④
Xuhua et al. (2013)	22	22	—	—	JH+Bio	Bio	3	①④⑤
Qing and Wengfang (2014)	40	40	—	—	FJH+Bio	Bio	6	①②⑤
Long et al. (2014)	50	50	—	—	QJLF+Bio	Bio	9	①④
Qianxin et al. (2014)	92	90	—	—	JH+Bio	Bio	2	①②⑤
Xiue et al. (2014)	97	98	31.6 ± 2.4	32.1 ± 2.1	QJLF+Bio	Bio	8	①②③⑥⑦
Jian and Li (2014)	24	24	68.5 ± 6.2	67.2 ± 5.7	JH+Bio	Bio	6	①⑥⑦
Jinghua et al. (2015)	53	53	21–73	20–73	KL+Bio	Bio	3	①
Xiang (2015)	50	58	22–87	23–88	KF+Bio	Bio	6	①④
Min-shuang et al. (2015)	56	58	42.18 ± 6.34	43.08 ± 6.63	JH+Bio	Bio	18	①⑤⑥⑦
Tianfeng (2015)	43	42	30–58	31–58	BFHX+Bio	Bio	3	①④
Yunxiao and Guosheng (2015)	40	40	—	—	BJ+Bio	Bio	6	①②③⑤
Yuxia (2015)	45	45	18–66	18–65	JH+Bio	Bio	2	①②⑤
Shuhua and Fujian (2016)	60	60	18–80	16–78	BL+Bio	Bio	6	①②
Shiming et al. (2016)	51	50	18–64	29–65	QJLF+Bio	Bio	18	①②③⑤
Qi et al. (2016)	150	150	—	—	BL+Bio	Bio	8	①②④⑤
Juan et al. (2016)	56	53	—	—	QJLF+Bio	Bio	8	①②③⑤
Wei (2016)	48	48	13–74	16–72	JPRF+Bio	Bio	6	①⑤
Zhenru (2016)	34	34	20–62	21–61	JH+Bio	Bio	6	②⑥⑦
Liping and Chun (2016)	42	28	—	—	FT+Bio	Bio	6	①②
Junguo et al. (2016)	40	38	18–65	17–64	JH+Bio	Bio	6	①②⑤
Qi (2016)	56	56	45.2 ± 3.4		JH+Bio	Bio	12	①②
Shuang (2016)	40	38	—	—	JHL+Bio	Bio	6	①②④
Yongyi et al. (2017)	55	55	19–66	17–67	JH+Bio	Bio	6	①②④⑤
Ji et al. (2017)	58	55	—	—	FT+Bio	Bio	6	①②③⑤
Xing et al. (2017)	185	175	—	—	JH+Bio	Bio	12	①②③⑤
Jun et al. (2017)	56	53	—	—	QJLF+Bio	Bio	8	①②③⑤
Xu et al. (2018)	90	90	45.6 ± 9.9	42.8 ± 10.5	BFHX+Bio	Bio	6	①②③
Liping (2018)	43	43	18.3–76.5	18.1–76.9	JH+Bio	Bio	6	①②⑤⑥⑦
Lingwei (2018)	60	60	—	—	JH+Bio	Bio	6	②④
Junjie (2018)	57	57	19–63	20–62	QJLF+Bio	Bio	9	①⑤
Xiaoru et al. (2018)	54	54	33.8 ± 5.7	33.7 ± 5.3	KF+Bio	Bio	6	①
Xiaoni (2018)	50	50	24–63	25–65	KL+Bio	Bio	6	①④
Di et al. (2019)	35	35	19–58	18–60	FJH+Bio	Bio	6	②
Zhibo et al. (2019)	68	67	35.9 ± 10.5	36.3 ± 10.1	KF+Bio	Bio	6	①②③⑤
Meng (2019)	40	40	13–74	12–75	JH+Bio	Bio	6	①⑤
Junmei et al. (2019)	41	41	22–68	21–69	BFHX+Bio	Bio	4	①⑤⑦
Hai-xian et al. (2019)	30	30	33–59	31–61	KF+Bio	Bio	6	①③⑤
Guizhen (2019)	55	50	25–66	29–63	JH+Bio	Bio	9	①③④⑤
Qin (2019)	53	53	24–77	23–78	FT+Bio	Bio	6	①②③⑤
Qingfeng (2019)	30	30	32–69	33–68	KL+Bio	Bio	6	①
Guannan et al. (2020)	158	158	72.87 ± 7.12	73.91 ± 7.37	KF+Bio	Bio	6	①③④⑤
Hui et al. (2020)	220	180	48.82 ± 6.94	49.46 ± 6.95	BL+Bio	Bio	12	①②③⑤⑦
Weiyong et al. (2020)	50	50	—	—	KL+Bio	Bio	6	①④
Xian-hui and Shu-lin (2020)	75	75	32–60	36–65	FT+Bio	Bio	6	①②③④⑤
Bin (2020)	50	50	25–76	22–75	JH+Bio	Bio	6	①②④⑤
Shuangli et al. (2020)	90	90	18–60	18–58	BL+Bio	Bio	3	①②③④⑤⑥⑦
Qiong et al. (2021)	101	101	17–75	16–72	JH+Bio	Bio	6	①
Wei-wei (2021)	62	62	60–81	60–76	JH+Bio	Bio	6	①⑥⑦
Qiang (2021)	60	60	49.14 ± 10.66	48.85 ± 10.27	JH+Bio	Bio	6	①②③④⑤
Qihuang (2022)	37	37	35–71	32–72	JH+Bio	Bio	3	①④⑤
Xuehu et al. (2022)	60	60	19–65	20–64	KF+Bio	Bio	3	①②③⑤
Yan et al. (2022)	56	56	32–67	30–68	JH+Bio	Bio	6	①②③④
Jia (2022)	50	50	25–67	24–69	JH+Bio	Bio	2	①
Jiafu and Lanhua (2022)	38	38	25–73	24–72	JH+Bio	Bio	6	②⑤
Xueyun (2022)	60	60	37.62 ± 6.91	38.15 ± 7.11	KF+Bio	Bio	6	①②③④⑤
Xiaohua (2022)	34	34	23–64	24–66	KF+Bio	Bio	6	①⑤⑥⑦
Juan (2022)	100	100	57.83 ± 5.09	52.17 ± 6.07	JH+Bio	Bio	9	①②③
Chenyong et al. (2022)	41	39	6–15	7–16	JPRF+Bio	Bio	3	①②③④⑤
Tao and Fei (2022)	40	40	26–75	24–75	JH+Bio	Bio	6	①②③④⑤
Yawei et al. (2023)	76	76	39–75	35–75	BFHX+Bio	Bio	6	①⑤
Guannan et al. (2023)	94	94	24–68	19–70	KF+Bio	Bio	6	①②③⑤
Qiaoe et al. (2023)	100	100	23–74	22–74	JH+Bio	Bio	6	①④
Junguo (2023)	30	30	70.48 ± 2.61	70.44 ± 2.23	JH+Bio	Bio	6	②⑤⑦
Yan et al. (2023)	35	35	55.61 ± 11.12	52.47 ± 10.49	KL+Bio	Bio	6	③⑥⑦
Yuxia (2023)	38	38	19–80	19–80	JH+Bio	Bio	6	①⑤
Hu et al. (2023)	68	68	41.87 ± 11.35	42.82 ± 11.02	BFHX+Bio	Bio	5	= 1 \* GB3 ①②③④⑤⑥⑦
Peiqian et al. (2024)	49	49	15–17	15–18	JH+Bio	Bio	6	⑤⑥⑦
Xinlei and Rong (2024)	104	103	49.44 ± 12.79	50 ± 15.01	JH+Bio	Bio	6	= 2 \* GB3 ②④⑤
Yuelian (2024)	49	49	52.97 ± 7.05	54.11 ± 6.83	JH+Bio	Bio	6	②③④⑥⑦

### 3.3 Literature quality assessment

**FIGURE 2:**
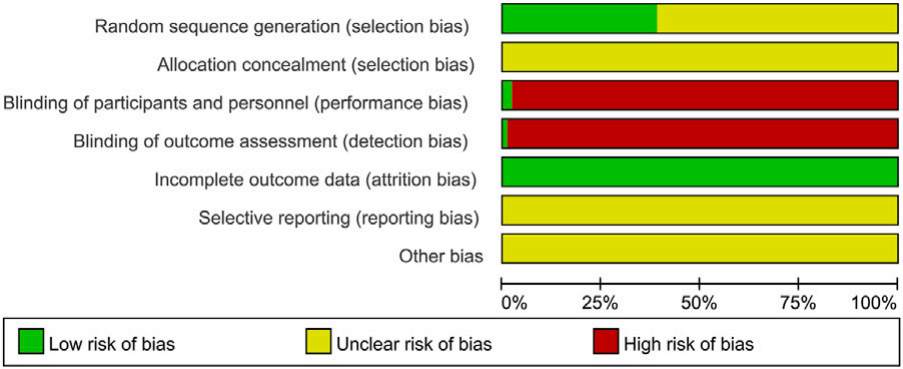
Percentages of items with risks of bias of included articles. In the domain of random sequence generation, 38 studies reported specific methods of randomization, while 62 studies merely mentioned randomization without providing details of the method used. For allocation concealment, none of the included studies reported any information. Regarding blinding of participants and personnel, one study reported a double-blind design, and another study adopted a single-blind design. In terms of blinding of outcome assessment, only one study reported using a double-blind approach. All included studies had complete outcome data. None of the studies mentioned selective reporting or other sources of bias. The term “other biases” refers to potential sources of bias such as funding-related bias, selective reporting bias, data collection bias, and study design bias.

All 100 included articles documented randomization procedures, with 28 employing random number tables (Mengqiu et al., 2006; Hualiang, 2013; Hunxi and Zhanqi, 2013; Xiue et al., 2014; Min-shuang et al., 2015; Juan et al., 2016; Zhenru, 2016; Ji et al., 2017; Jun et al., 2017; Xiaoru et al., 2018; Junmei et al., 2019; Hai-xian et al., 2019; Bin, 2020; Guannan et al., 2020; Weiyong et al., 2020; Qiang, 2021; Qihuang, 2022; Xiaohua, 2022; Xueyun, 2022; Guannan et al., 2023; Chuanjun et al., 2023; Qiaoe et al., 2023; Chenyong et al., 2022; Yawei et al., 2023; Yuxia, 2023; Peiqian et al., 2024; Xinlei and Rong, 2024; Yuelian, 2024), one utilizing random pairing (Jun, 2010), one applying random ordering (Wen-ge et al., 2013), two implementing random sampling (Juan et al., 2016; Jun et al., 2022), one adopting randomized controlled design (Chang et al., 2022), one using stratified block randomization (Guannan et al., 2020), two applying computer randomization (Xian-hui and Shu-lin, 2020; Juan, 2022); one implementing random parallel design (Chenyong et al., 2022), one using pathogen number draw (Yongyi et al., 2017), all rated as low risk. Studies mentioning randomization without methodological specification received unclear risk ratings. The investigation by Liu Xing et al. (Xing et al., 2017) implemented double-blind method and was rated as low risk, whereas Gao Mengqiu et al. (Mengqiu et al., 2006) applied single-blind method without specifying personnel responsible for implementation. All the included studies had complete data, with no evidence of selective reporting or other biases documented. Detailed risk of bias assessments are presented in Figure 2.

### 3.4 Evidence network

**FIGURE 3:**
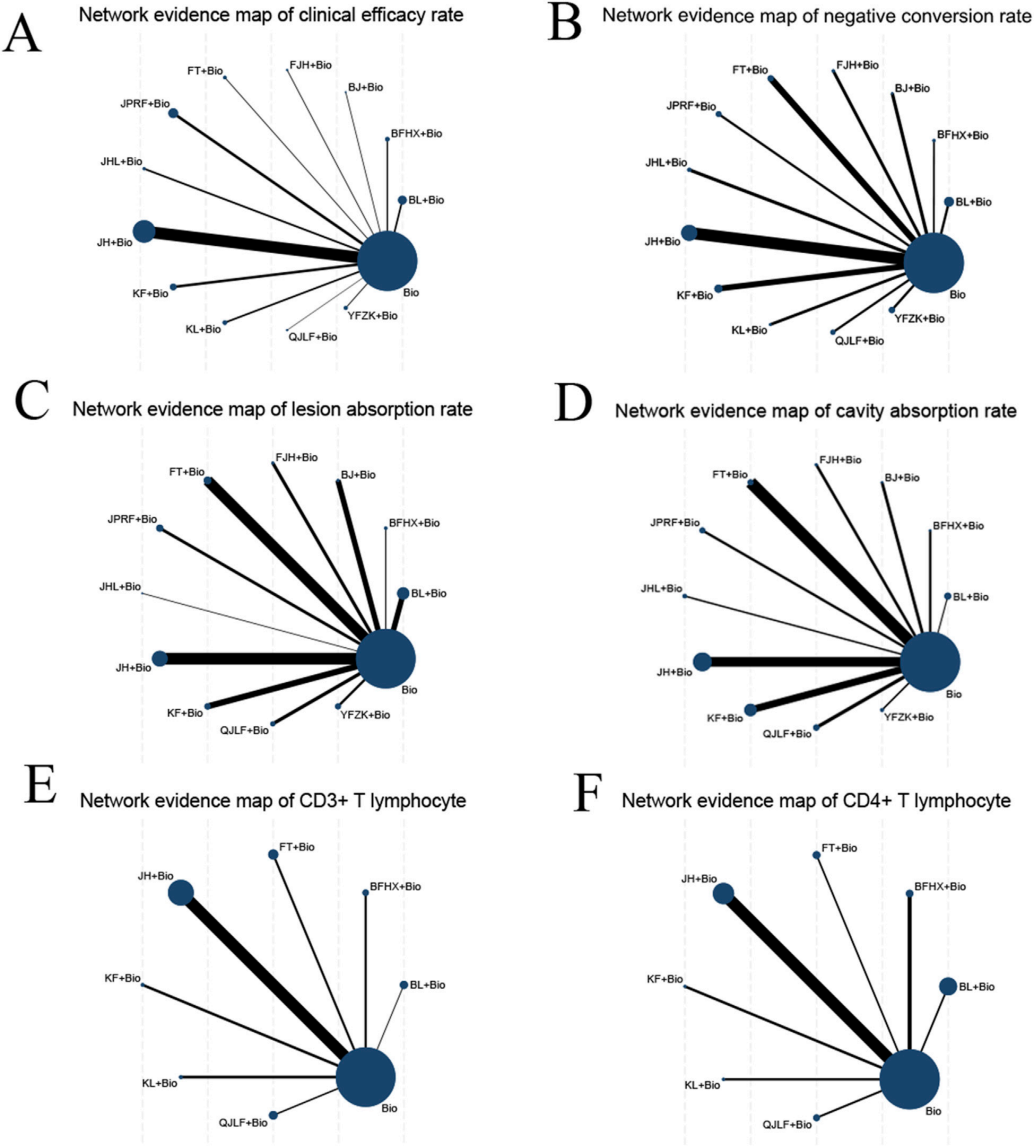
Network evidence maps comparing different outcomes: **(A)** network evidence map for clinical efficacy rate; **(B)** network evidence map for negative conversion rate; **(C)** network evidence map for lesion absorption rate; **(D)** network evidence map for cavity absorption rate; **(E)** network evidence map for improvement rate of CD3^+^ T lymphocytes; **(F)** network evidence map for improvement rate of CD4^+^ T lymphocytes.

Fifty-three studies reported clinical efficacy rates, resulting in 12 direct comparisons, while 78 studies reported negative conversion rates yielding 12 direct comparisons. Evaluation of lesion absorption rates occurred across 56 studies generating 11 direct comparisons., with cavity absorption rates assessed in 42 studies forming 11 direct comparisons. 13 studies quantified CD3^+^ T lymphocyte improvement rates forming seven direct comparisons, complemented by 16 studies examining CD4^+^ T lymphocyte improvement rates forming seven direct comparisons. No closed loops were formed between the outcome indicators, so inconsistency tests were not conducted, with the complete evidence network structure depicted in Figure 3.

### 3.5 Network meta-analysis

#### 3.5.1 Clinical efficacy rate

**FIGURE 4:**
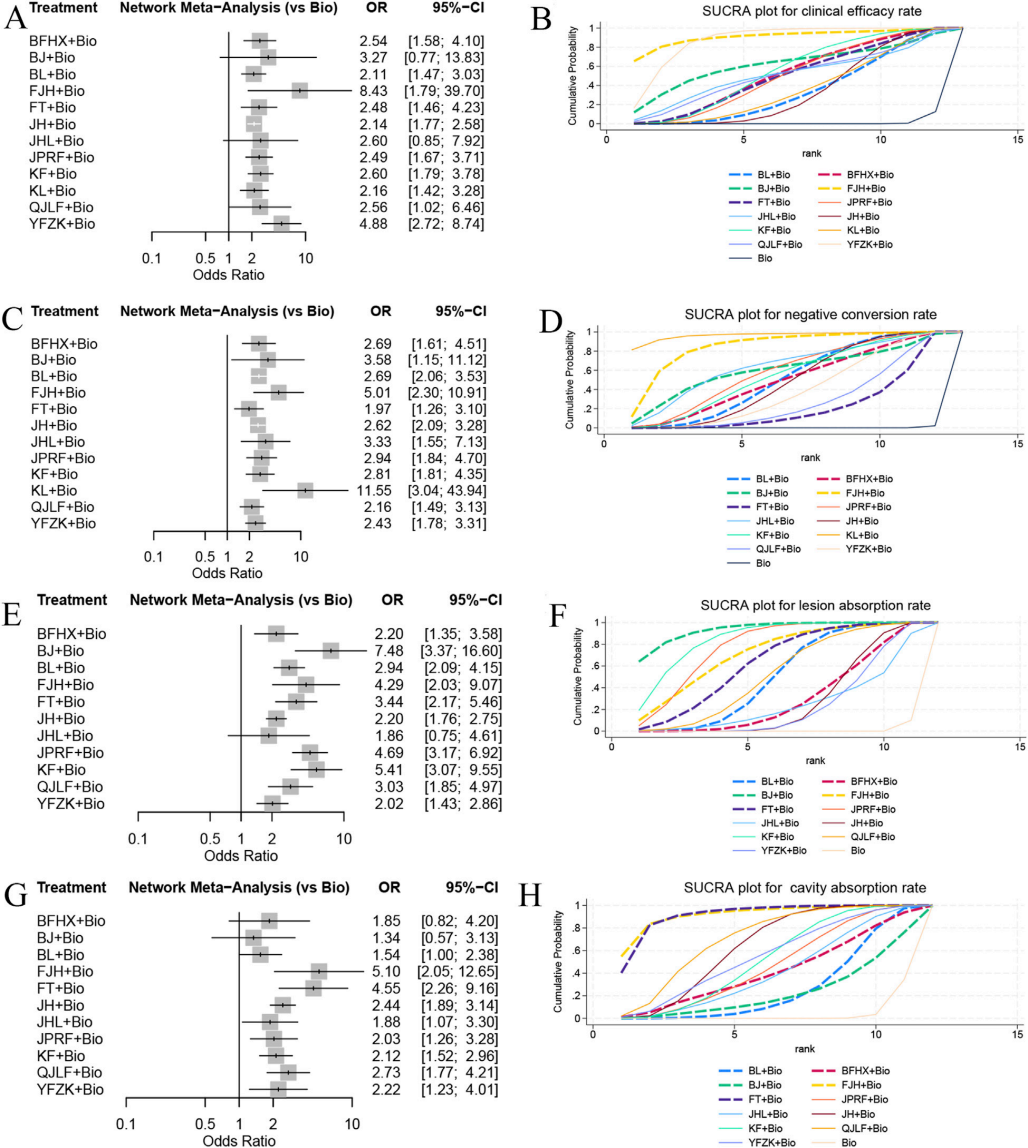
Forest plots and SUCRA ranking charts showing comparative effects on clinical efficacy rate, negative conversion rate, lesion absorption rate, and cavity absorption rate: **(A)** Forest plot of clinical efficacy rate; **(B)** SUCRA ranking chart for clinical efficacy rate; **(C)** Forest plot of negative conversion rate; **(D)** SUCRA ranking chart for negative conversion rate; **(E)** Forest plot of lesion absorption rate; **(F)** SUCRA ranking chart for lesion absorption rate; **(G)** Forest plot of cavity absorption rate; **(H)** SUCRA ranking chart for cavity absorption rate.

Assessment of clinical efficacy across 53 studies reported clinical efficacy rate, incorporating 13 interventions, including 12 types of oral TCMs, enrolled 6,625 participants. The network meta-analysis demonstrated significantly enhanced clinical efficacy for combinations of standard biomedicine with: FeiJieHe Pill (OR = 8.43,95% CI [1.79, 39.7]), Yi Fei Zhi Ke Capsule (OR = 4.88, 95% CI [2.72, 8.74]), Kangfuxin Liquid (OR = 2.60, 95% CI [1.79, 3.78]), Qi Jia Li Fei Capsule (OR = 2.56, 95% CI [1.02, 6.46]), Bu Fei Huo Xue Capsule (OR = 2.54, 95% CI [1.58, 4.10]), Jian Pi Run Fei Pill (OR = 2.49, 95% CI [1.67, 3.71]), Fei Tai Capsule (OR = 2.48, 95% CI [1.46, 4.23]), KangLao Pill (OR = 2.16, 95% CI [1.42, 3.28]), Bai Ling Capsule (OR = 2.11, 95% CI [1.47, 3.03]), and Jie He Pill (OR = 2.14, 95% CI [1.77, 2.58]), all relative to biomedicine monotherapy. SUCRA ranking for clinical efficacy revealed the following hierarchy of therapeutic combinations: FeiJieHe Pill with standard biomedicine (SUCRA = 90.9%) exhibited the highest probability of superiority, followed sequentially by Yi Fei Zhi Ke Capsule (SUCRA = 87.2%), Bu Jin Tablet (SUCRA = 61.5%), Kangfuxin Liquid (SUCRA = 54.3%), Bu Fei Huo Xue Capsule (SUCRA = 51.3%), Jie He Ling Tablet (SUCRA = 50.7%), Qi Jia Li Fei Capsule (SUCRA = 50.3%), Jian Pi Run Fei Pill (SUCRA = 49.7%), Fei Tai Capsule (SUCRA = 49.2%), KangLao Pill (SUCRA = 36.2%), Bai Ling Capsule (SUCRA = 34%), and Jie He Pill (SUCRA = 33.7%), with all relative to standard biomedicine monotherapy (SUCRA = 1.1%). In summary, the combination of FeiJieHe Pill with standard biomedicine potentially represents the optimal approach for improving clinical efficacy, as visually summarized in Figures 4A,B.

#### 3.5.2 Negative conversion rate

A total of 78 studies reported the negative conversion rate, involving 13 interventions, including 12 types of oral TCMs, with 9,955 participants. The network meta-analysis results demonstrated significantly greater negative conversion rate for combination of standard biomedicine with: KangLao Pill (OR = 11.55, 95% CI [3.04, 43.93]), FeiJieHe Pill (OR = 5.01, 95% CI [2.30,10.91]), Jie He Ling Tablet (OR = 3.33, 95% CI [1.55, 7.13]), Bu Jin Tablet (OR = 3.58, 95% CI [1.15,11.12]), Jian Pi Run Fei Pill (OR = 2.94, 95% CI [1.84, 4.70]), Kangfuxin Liquid (OR = 2.81, 95% CI [1.81, 4.35]), Bai Ling Capsule (OR = 2.69, 95% CI [2.06, 3.53]), Bu Fei Huo Xue Capsule (OR = 2.69, 95% CI [1.61, 4.51]), Jie He Pill (OR = 2.62, 95% CI [2.09, 3.28]), Yi Fei Zhi Ke Capsule (OR = 2.43, 95% CI [1.78, 3.31]), Qi Jia Li Fei Capsule (OR = 2.16, 95% CI [1.49, 3.13]), and Fei Tai Capsule (OR = 1.97, 95%CI [1.26, 3.10]), all relative to standard biomedicine monotherapy. SUCRA ranking for negative conversion rate established a probabilistic hierarchy wherein KangLao Pill combined with standard biomedicine (SUCRA = 96.5%) showed the highest superiority probability, followed sequentially by FeiJieHe Pill (SUCRA = 84.2%), Jie He Ling Tablet (SUCRA = 63.3%), Bu Jin Tablet (SUCRA = 59.7%), Jian Pi Run Fei Pill (SUCRA = 57.4%), Kangfuxin Liquid (SUCRA = 53.8%), Bai Ling Capsule (SUCRA = 50.2%), Bu Fei Huo Xue Capsule (SUCRA = 49.2%), Jie He Pill (SUCRA = 47.3%), Yi Fei Zhi Ke Capsule (SUCRA = 38.7%), Qi Jia Li Fei Capsule (SUCRA = 28%), and Fei Tai Capsule (SUCRA = 21.6%), all benchmarked against standard biomedicine monotherapy (SUCRA = 0.2%). In summary, KangLao Pill combined with standard biomedicine potentially represents the optimal approach for improving negative conversion rates, as visualized in Figures 4C,D.

#### 3.5.3 Lesion absorption rate

A total of 56 studies reported lesion absorption rate, involving 12 interventions, including 11 types of oral TCMs, with a total of 7,676 patients. The network meta-analysis showed a higher lesion absorption rate for the combinations of standard biomedicine with: Bu Jin Tablet (OR = 7.48, 95% CI [3.37,16.60]), Kangfuxin Liquid (OR = 5.41, 95% CI [3.07, 9.55]), Jian Pi Run Fei Pill (OR = 4.69, 95% CI [3.17, 6.62]), FeiJieHe Pill (OR = 4.29, 95% CI [2.03, 9.07]), Fei Tai Capsule (OR = 3.44, 95% CI [2.17, 5.46]), Qi Jia Li Fei Capsule (OR = 3.03, 95% CI [1.85, 4.97]), Bai Ling Capsule (OR = 2.94, 95% CI [2.09, 4.15]), Bu Fei Huo Xue Capsule (OR = 2.20, 95% CI [1.35,3.58]), Jie He Pill (OR = 2.20, 95% CI [1.76, 2.75]), and Yi Fei Zhi Ke Capsule (OR = 2.02, 95% CI [1.43, 2.86]), than using biomedicine monotherapy. SUCRA ranking for clinical efficacy revealed the following hierarchy of therapeutic combinations of Bu Jin Tablet with standard biomedicine (SUCRA = 93.4%) exhibited the highest lesion absorption rate, followed sequentially by Kangfuxin Liquid (SUCRA = 84.5%), Jian Pi Run Fei Pill (SUCRA = 77.3%), FeiJieHe Pill (SUCRA = 71.5%), Fei Tai Capsule (SUCRA = 63.5%), Qi Jia Li Fei Capsule (SUCRA = 52.3%), Bai Ling Capsule (SUCRA = 50%), Bu Fei Huo Xue Capsule (SUCRA = 30%), Jie He Pill (SUCRA = 23.7%), Jie He Ling Tablet (SUCRA = 25.3%), Yi Fei Zhi Ke Capsule (SUCRA = 24.1%) all relative to standard biomedicine monotherapy (SUCRA = 0.9%). In summary, the combination of Bu Jin Tablet with standard biomedicine may be the best approach for improving lesion absorption rate, as visually summarized in Figures 4E,F.

#### 3.5.4 Cavity absorption rate

A total of 42 studies reported cavity absorption rate, involving 12 interventions, including 11 types of TCMs, enrolled 5,148 patients. The network meta-analysis results showed significantly greater cavity absorption rate for combination of standard biomedicine with: FeiJieHe Pill (OR = 5.10, 95% CI [2.05, 12.65]), Fei Tai Capsule (OR = 4.55, 95% CI [2.26, 9.16]), Qi Jia Li Fei Capsule (OR = 2.73, 95% CI [1.77, 4.21]), Jie He Pill (OR = 2.44, 95% CI [1.89, 3.14]), Yi Fei Zhi Ke Capsule (OR = 2.22, 95% CI [1.23, 4.01]), Kangfuxin Liquid (OR = 2.12, 95% CI [1.52, 2.96]), Jian Pi Run Fei Pill (OR = 2.03, 95% CI [1.26, 3.28]), and Jie He Ling Tablet (OR = 1.88, 95% CI [1.07, 3.30]), all relative to biomedicine monotherapy. SUCRA ranking for cavity absorption rate established a probabilistic hierarchy wherein FeiJieHe Pill combined with standard biomedicine (SUCRA = 91.9%) showed the highest superiority probability, followed by Fei Tai Capsule (SUCRA = 91.1%), Qi Jia Li Fei Capsule (SUCRA = 69.9%), Jie He Pill (SUCRA = 62.7%), Yi Fei Zhi Ke Capsule (SUCRA = 53.5%), Kangfuxin Liquid (SUCRA = 51.1%), Jian Pi Run Fei Pill (SUCRA = 46.1%), Jie He Ling Tablet (SUCRA = 41.2%), Bu Fei Huo Xue Capsule (SUCRA = 40.5%), Bai Ling Capsule (SUCRA = 26.1%), Bu Jin Tablet (SUCRA = 22.4%), all benchmarked against standard biomedicine monotherapy (SUCRA = 3.4%). In summary, the combination of FeiJieHe Pill with standard biomedicine may be the best approach for improving cavity absorption rate, as visualized in Figures 4G,H.

#### 3.5.5 CD3^+^ T lymphocyte and CD4^+^ T lymphocyte improvement rate

**FIGURE 5:**
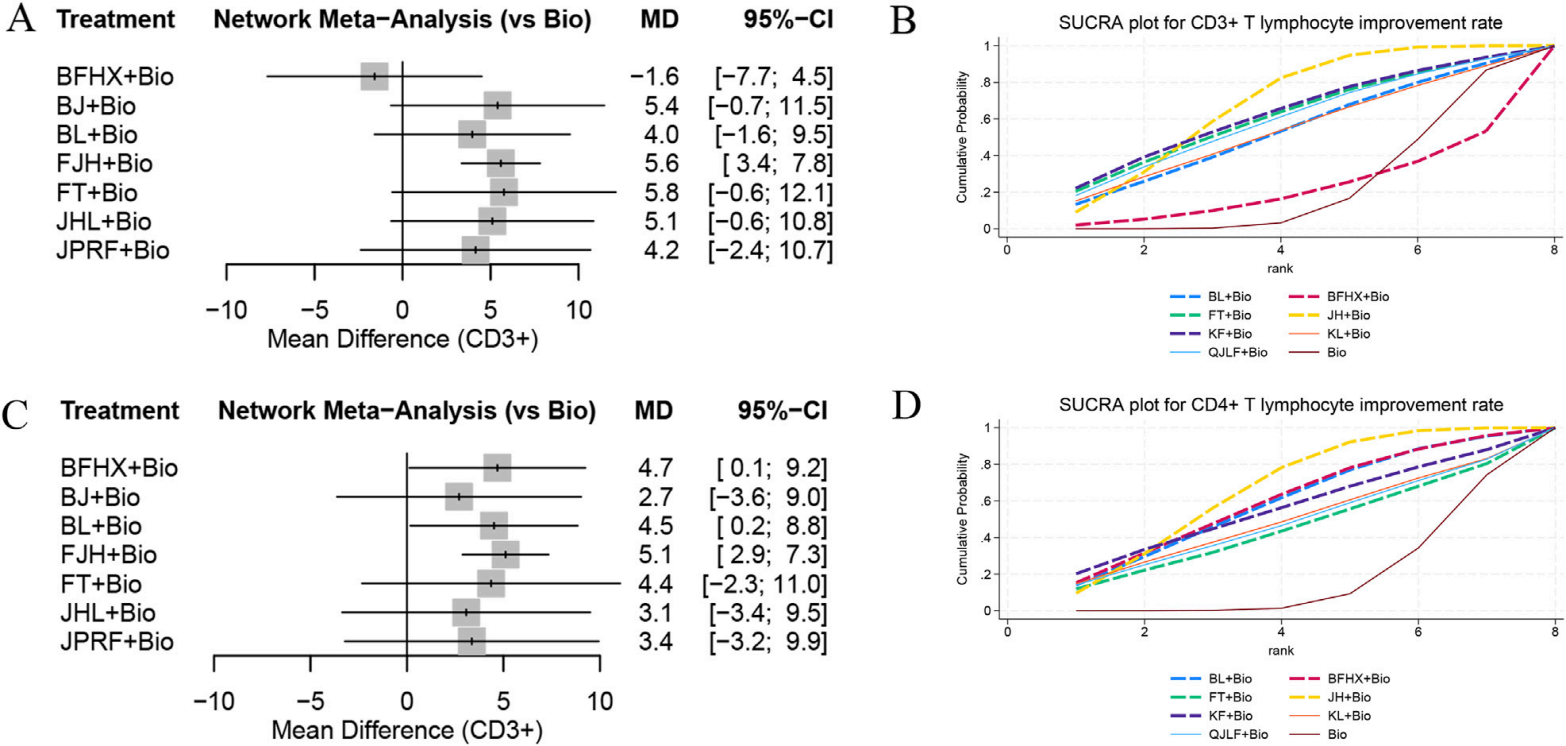
Forest plots and SUCRA ranking charts for CD3^+^ T lymphocyte improvement rate and CD4^+^ T lymphocyte improvement rate: **(A)** forest plot of CD3^+^ T lymphocyte improvement rate; **(B)** SUCRA ranking chart for CD3^+^ T lymphocyte improvement rate; **(C)** forest plot of CD4^+^ T lymphocyte improvement rate; **(D)** SUCRA ranking chart for CD4^+^ T lymphocyte improvement rate.

A total of 13 studies involving 1,519 participants across eight interventions, comprising seven types of oral TCMs, reported CD3^+^ T lymphocyte improvement rates. The network meta-analysis revealed superior improvement rate of CD3^+^ T lymphocytes for JieHe pill combined with conventional biomedicine (OR = 5.6, 95% CI [3.4, 7.8]) than monotherapy. Separately, 16 studies encompassing 2,057 participants under eight interventions (including seven oral TCM types) documented CD4^+^ T lymphocyte improvement rate. The network meta-analysis results show that the improvement rate of CD4^+^ T lymphocytes was significantly enhanced outcomes for the combination of conventional biomedicine with JieHe pill (OR = 5.1, 95% CI [2.9, 7.3]), Bu Fei Huo Xue Capsule (OR = 4.7, 95% CI [0.1, 9.2]), and Bai Ling Capsule (OR = 4.5, 95% CI [0.2, 8.8]) compared to biomedicine monotherapy. SUCRA ranking for CD3^+^ T lymphocyte improvement rate indicated the following hierarchy: the combination of standard biomedicine with Jie He Pill demonstrated superiority over Kangfuxin Liquid combination therapy (SUCRA = 67.6%), followed by Kangfuxin Liquid (SUCRA = 63.5%), Fei Tai Capsule (SUCRA = 60.7%), Qi Jia Li Fei Capsule (SUCRA = 59.2%), KangLao Pill (SUCRA = 53%), Bai Ling Capsule (SUCRA = 52.8%), standard biomedicine monotherapy (SUCRA = 22%), and Bu Fei Huo Xue Capsule (SUCRA = 21.3%), as shown in Figures 5A,B. For CD4^+^ T lymphocyte improvement rate, the probability ranking positioned Jie He Pill combined with standard biomedicine (SUCRA = 66.5%) first, followed by Bu Fei Huo Xue Capsule (SUCRA = 60.4%), Bai Ling Capsule (SUCRA = 58.7%), Kangfuxin Liquid (SUCRA = 56.3%), KangLao Pill (SUCRA = 48.9%), Qi Jia Li Fei Capsule (SUCRA = 47.1%), and Fei Tai Capsule (SUCRA = 44.9%), each outperforming standard biomedicine monotherapy (SUCRA = 17.2%), as shown in Figures 5C,D.

### 3.6 Adverse reactions

Safety assessment across 61 encompassing 8,573 participants (4,496 in the experimental group and 4,077 in the control group) The majority of adverse reactions in both groups were related to the digestive system, characterized by mild, self-limiting manifestations. The experimental group reported 586 adverse reactions (approximate incidence 13%), compared with 1,022 adverse reactions (approximate incidence 25%) in controls. Since the types of adverse reactions reported in the included studies varied, only a descriptive analysis is provided here, as detailed in Table 2.

**TABLE 2:** Adverse reactions reported in the included randomized controlled trials.

Included in study	Interventions		Adverse reactions	
	T	C	T	C
Junmei et al. (2019)	BFHX+Bio	Bio	2 cases of gastrointestinal reactions, 1 case of skin allergy, 1 case of gout, and 2 cases of liver function impairment.	2 cases of gastrointestinal reactions, 2 cases of skin allergies, 1 case of gout, and 3 cases of liver function impairment.
Yawei et al. (2023)	BFHX+Bio	Bio	3 cases of dizziness, 2 cases of liver function impairment, 5 cases of gastrointestinal reactions, 1 case of peripheral neuritis, 2 cases of rash.	2 cases of dizziness, 1 case of liver function impairment, 6 cases of gastrointestinal reactions, and 2 cases of rash.
Hu (2023)	BFHX+Bio	Bio	5 cases of abnormal liver function, and 2 cases of dry mouth.	10 cases of abnormal liver function, and 6 cases of gastrointestinal reactions.
Yunxiao and Guosheng (2015)	BJ+Bio	Bio	2 cases of abnormal liver function.	5 cases of abnormal liver function.
Yanpei and Shuyu (2002)	BL+Bio	Bio	9 cases of adverse reactions.	17 cases of adverse reactions.
Ge et al. (2009)	BL+Bio	Bio	12 cases of abnormal liver function.	31 cases of abnormal liver function.
Qi et al. (2016)	BL+Bio	Bio	15 cases of abnormal liver function, 1 case of kidney function injury, 13 cases of dizziness and tinnitus, and 7 cases of blood system reactions.	17 cases of abnormal liver function, 7 cases of kidney function injury, 16 cases of dizziness and tinnitus, and 19 cases of blood system reactions.
Hui et al. (2020)	BL+Bio	Bio	29 cases of gastrointestinal reactions, 3 cases of abnormal kidney function, 5 cases of abnormal liver function, 9 cases of hypothyroidism, 10 cases of blood system abnormalities, and 7 cases of neurological dysfunction.	35 cases of gastrointestinal reactions, 12 cases of abnormal kidney function, 16 cases of abnormal liver function, 13 cases of hypothyroidism, 22 cases of blood system abnormalities, and 12 cases of neurological dysfunction.
Shuangli et al. (2020)	BL+Bio	Bio	11 cases of gastrointestinal reactions, 1 case of abnormal kidney function, 2 cases of abnormal liver function, 4 cases of blood system injuries, and 3 cases of neurological injuries.	17 cases of gastrointestinal reactions, 6 cases of abnormal kidney function, 8 cases of abnormal liver function, 11 cases of blood system injuries, and 6 cases of neurological injuries.
Fengyan (2012)	FJH+Bio	Bio	2 cases of abnormal liver function, and 1 case of gastrointestinal reaction.	3 cases of abnormal liver function.
Qing and Wenfang (2014)	FJH+Bio	Bio	2 cases of abnormal liver function.	3 cases of abnormal liver function.
Mengqiu et al. (2006)	FT+Bio	Bio	1 case of elevated transaminase levels, and 1 case of Achilles tendon pain caused by levofloxacin.	Not reported.
Ji et al. (2017)	FT+Bio	Bio	3 cases of gastrointestinal reactions, 1 case of allergic reaction, 4 cases of liver injury, 3 cases of leukopenia (reduced white blood cells), 2 cases of gout, and 1 case of other conditions.	3 cases of gastrointestinal reactions, 2 cases of allergic reactions, 5 cases of liver injury, 4 cases of leukopenia (reduced white blood cells), 1 case of gout, and 1 case of other conditions.
Qingfeng (2019)	FT+Bio	Bio	2 cases of gastrointestinal reactions, 3 cases of functional impairment, 2 cases of leukopenia (reduced white blood cells), 1 case of gout, and 1 case of other conditions.	3 cases of gastrointestinal reactions, 6 cases of functional impairment, 5 cases of leukopenia (reduced white blood cells), 2 cases of gout, and 1 case of other conditions.
Xian-hui and Shu-lin (2020)	FT+Bio	Bio	4 cases of gastrointestinal reactions, 1 case of allergic reaction, 5 cases of abnormal liver function, 4 cases of leukopenia, 2 cases of gout.	3 cases of gastrointestinal reactions, 3 cases of allergic reactions, 4 cases of abnormal liver function, 3 cases of leukopenia (reduced white blood cells), and 1 case of gout.
Lidong and Shuqing (2007)	JHL+Bio	Bio	1 case of mildly abnormal liver function, and 2 cases of gastrointestinal reactions.	Not reported.
Shuqin et al. (2008)	JHL+Bio	Bio	2 cases of mild liver function impairment.	Not reported.
Zhongming (2013)	JHL+Bio	Bio	1 case of gastrointestinal reaction, and 1 case of liver function impairment.	Not reported.
Guiqiu (2010)	JH+Bio	Bio	2 cases of gastrointestinal reactions, and 1 case of liver function impairment.	Not reported.
Dan et al. (2013)	JH+Bio	Bio	6 cases of adverse reactions.	7 cases of adverse reactions.
Xuhua et al. (2013)	JH+Bio	Bio	4 cases of gastrointestinal reactions, 1 case of abnormal liver function, and 1 case of abnormal kidney function.	8 cases of gastrointestinal reactions, 5 cases of abnormal liver function, 1 case of abnormal kidney function, and 2 cases of rash.
Qianxin et al. (2014)	JH+Bio	Bio	6 cases of abnormal liver function.	19 cases of abnormal liver function.
Min-shuang et al. (2015)	JH+Bio	Bio	3 cases of gastrointestinal reactions.	5 cases of gastrointestinal reactions.
Yuxia (2015)	JH+Bio	Bio	1 case of abnormal liver function.	5 cases of abnormal liver function.
Junguo et al. (2016)	JH+Bio	Bio	1 case of abnormal liver function.	4 cases of abnormal liver function.
Yongyi et al. (2017)	JH+Bio	Bio	3 cases of abnormal liver function.	6 cases of abnormal liver function.
Xing et al. (2017)	JH+Bio	Bio	5 cases of adverse reactions.	8 cases of adverse reactions.
Liping (2018)	JH+Bio	Bio	1 case of abnormal liver function, 2 cases of gastrointestinal reactions, and 1 case of abnormal kidney function.	2 cases of abnormal liver function, 4 cases of gastrointestinal reactions, and 3 cases of abnormal kidney function.
Meng (2019)	JH+Bio	Bio	2 cases of gastrointestinal reactions, and 1 case of abnormal liver function.	4 cases of gastrointestinal reactions, 3 cases of abnormal liver function, and 3 cases of abnormal kidney function.
Guizhen (2019)	JH+Bio	Bio	3 cases of leukopenia (reduced white blood cells), 7 cases of gastrointestinal reactions, and 6 cases of liver function impairment.	10 cases of leukopenia (reduced white blood cells), 18 cases of gastrointestinal reactions, 16 cases of liver function impairment, and 6 cases of joint pain.
Bin (2020)	JH+Bio	Bio	2 cases of gastrointestinal reactions, and 1 case of joint pain.	2 cases of gastrointestinal reactions, 1 case of joint pain, and 1 case of abnormal liver and kidney function.
Qiang (2021)	JH+Bio	Bio	5 cases of abnormal liver function, 11 cases of gastrointestinal reactions, 5 cases of allergic reactions, 2 cases of vision impairment, and 3 cases of other reactions.	4 cases of abnormal liver function, 13 cases of gastrointestinal reactions, 4 cases of allergic reactions, 3 cases of vision impairment, and 2 cases of other reactions.
Qihuang (2022)	JH+Bio	Bio	5 cases of gastrointestinal reactions, and 1 case of neuritis.	2 cases of gastrointestinal reactions, and 1 case of neuritis.
Jiafu and Lanhua (2022)	JH+Bio	Bio	1 case of gastrointestinal reaction, and 1 case of headache.	3 cases of gastrointestinal reactions, and 2 cases of headaches.
Tao and Fei (2022)	JH+Bio	Bio	3 cases of abnormal liver function, 7 cases of gastrointestinal reactions, 3 cases of allergic reactions, and 1 case of vision impairment.	2 cases of abnormal liver function, 8 cases of gastrointestinal reactions, 3 cases of allergic reactions, and 2 cases of vision impairment.
Junguo (2023)	JH+Bio	Bio	2 cases of gastrointestinal issues, and 1 case of abnormal liver and kidney function.	3 cases of gastrointestinal issues, and 1 case of dizziness.
Yuxia (2023)	JH+Bio	Bio	5 cases of gastrointestinal reactions, 1 case of abnormal liver function, and 2 cases of peripheral neuritis.	5 cases of gastrointestinal reactions, 2 cases of abnormal liver function, 1 case of reduced vision, and 3 cases of peripheral neuritis.
Peiqian et al. (2024)	JH+Bio	Bio	2 cases of gastrointestinal reactions, 1 case of liver and kidney injury, 1 case of dizziness, and 1 case of rash.	2 cases of gastrointestinal reactions, and 1 case of rash.
Xinlei and Rong (2024)	JH+Bio	Bio	3 cases of gastrointestinal dysfunction, 1 case of liver function impairment, 1 case of neurological damage, and 2 cases of rash.	15 cases of gastrointestinal discomfort, 2 cases of liver function impairment, 3 cases of neurological damage, and 7 cases of rash.
Wulu et al. (2007)	JPRF+Bio	Bio	4 cases of mild liver function impairment, and 2 cases of gastrointestinal reactions.	A total of 5 cases including liver function impairment, gastrointestinal reactions, neurological symptoms, and leukopenia (reduced white blood cells).
Shouyue (2009)	JPRF+Bio	Bio	12 cases of adverse reactions.	69 cases of adverse reactions.
Fuyuan and Meimingxing (2010)	JPRF+Bio	Bio	4 cases of mild liver function impairment, and 2 cases of gastrointestinal reactions.	4 cases of severe side effects, and 14 cases of mild side effects.
Bing and Yuanai (2011)	JPRF+Bio	Bio	1 case of allergic reaction, 7 cases of gastrointestinal reactions, 10 cases of abnormal liver function, and 1 case of abnormal urinalysis.	3 cases of allergic reactions, 23 cases of gastrointestinal reactions, 29 cases of abnormal liver function, and 4 cases of abnormal urinalysis.
Hunxi and Zhanqi (2013)	JPRF+Bio	Bio	9 cases of adverse reactions.	26 cases of adverse reactions.
Wei (2016)	JPRF+Bio	Bio	4 cases of adverse reactions.	11 cases of adverse reactions.
Chenyong et al. (2022)	JPRF+Bio	Bio	1 case of gastrointestinal reaction, 1 case of peripheral neuritis, and 1 case of rash.	2 cases of gastrointestinal reactions, 1 case of peripheral neuritis, 3 cases of rash, and 2 cases of abnormal liver function.
Wen-ge et al. (2013)	KF+Bio	Bio	5 cases of gastrointestinal reactions, 4 cases of abnormal liver function, and 2 cases of rash.	6 cases of gastrointestinal reactions, 4 cases of abnormal liver function, and 2 cases of rash.
Zhibo et al. (2019)	KF+Bio	Bio	10 cases of adverse reactions.	12 cases of adverse reactions.
Hai-xian et al. (2019)	KF+Bio	Bio	5 cases of elevated uric acid, 3 cases of liver function impairment, 3 cases of gastrointestinal reactions, and 1 case of knee joint pain.	3 cases of elevated uric acid, 4 cases of liver function impairment, 10 cases of gastrointestinal reactions, and 1 case of knee joint pain.
Guannan et al. (2020)	KF+Bio	Bio	9 cases of leukopenia (reduced white blood cells), 6 cases of gastrointestinal reactions, 6 cases of abnormal liver function, and 5 cases of elevated bilirubin levels.	8 cases of leukopenia (reduced white blood cells), 11 cases of gastrointestinal reactions, 6 cases of abnormal liver function, and 5 cases of elevated bilirubin levels.
Xuehu et al. (2022)	KF+Bio	Bio	4 cases of gastrointestinal reactions.	Not reported.
Xueyun (2022)	KF+Bio	Bio	1 case of allergic reaction, 1 case of liver injury, 1 case of gastrointestinal reaction, and 3 cases of gout.	2 cases of allergic reactions, 2 cases of liver injuries, 3 cases of gastrointestinal reactions, and 3 cases of gout.
Xiaohua (2022)	KF+Bio	Bio	1 case of gastrointestinal reaction, and 1 case of thrombocytopenia (reduced platelets).	4 cases of gastrointestinal reactions, 2 cases of leukopenia (reduced white blood cells), 2 cases of thrombocytopenia (reduced platelets), and 1 case of abnormal liver function.
Guannan et al. (2023)	KF+Bio	Bio	1 case of gastrointestinal reaction, 1 case of allergic reaction, 2 cases of liver injuries, 2 cases of leukopenia (reduced white blood cells), and 1 case of gout.	8 cases of gastrointestinal reactions, 3 cases of allergic reactions, 2 cases of liver injuries, 3 cases of leukopenia (reduced white blood cells), and 2 cases of gout.
Shiming et al. (2016)	QJLF+Bio	Bio	5 cases of abnormal liver function, 5 cases of gastrointestinal reactions, and 1 case of hypokalemia (low blood potassium).	4 cases of abnormal liver function, 4 cases of gastrointestinal reactions, 2 cases of dizziness, and 2 cases of tinnitus.
Juan et al. (2016)	QJLF+Bio	Bio	2 cases of gastrointestinal reactions, 10 cases of elevated uric acid, 2 cases of abnormal liver function, and 1 case of joint pain.	8 cases of elevated uric acid, and 3 cases of abnormal liver function.
Jun et al. (2017)	QJLF+Bio	Bio	2 cases of gastrointestinal reactions, 10 cases of elevated uric acid, 2 cases of abnormal liver function, and 1 case of joint pain.	8 cases of elevated uric acid, and 3 cases of abnormal liver function.
Junjie (2018)	QJLF+Bio	Bio	4 cases of elevated uric acid, 2 cases of abnormal liver function, and 3 cases of gastrointestinal reactions.	2 cases of elevated uric acid, 2 cases of abnormal liver function, and 2 cases of joint pain.
Xuewen (2007)	YFZK+Bio	Bio	8 cases of mild liver function impairment, and 5 cases of gastrointestinal reactions.	7 cases of mild liver function impairment, and 5 cases of gastrointestinal reactions.
Yujuan (2007)	YFZK+Bio	Bio	10 cases of adverse reactions.	12 cases of adverse reactions.
Xiuxia (2009)	YFZK+Bio	Bio	10 cases of gastrointestinal reactions, 8 cases of liver function impairment, and 5 cases of rash.	38 cases of gastrointestinal reactions, 16 cases of liver function impairment, and 4 cases of rash.

### 3.7 Publication bias assessment

**FIGURE 6:**
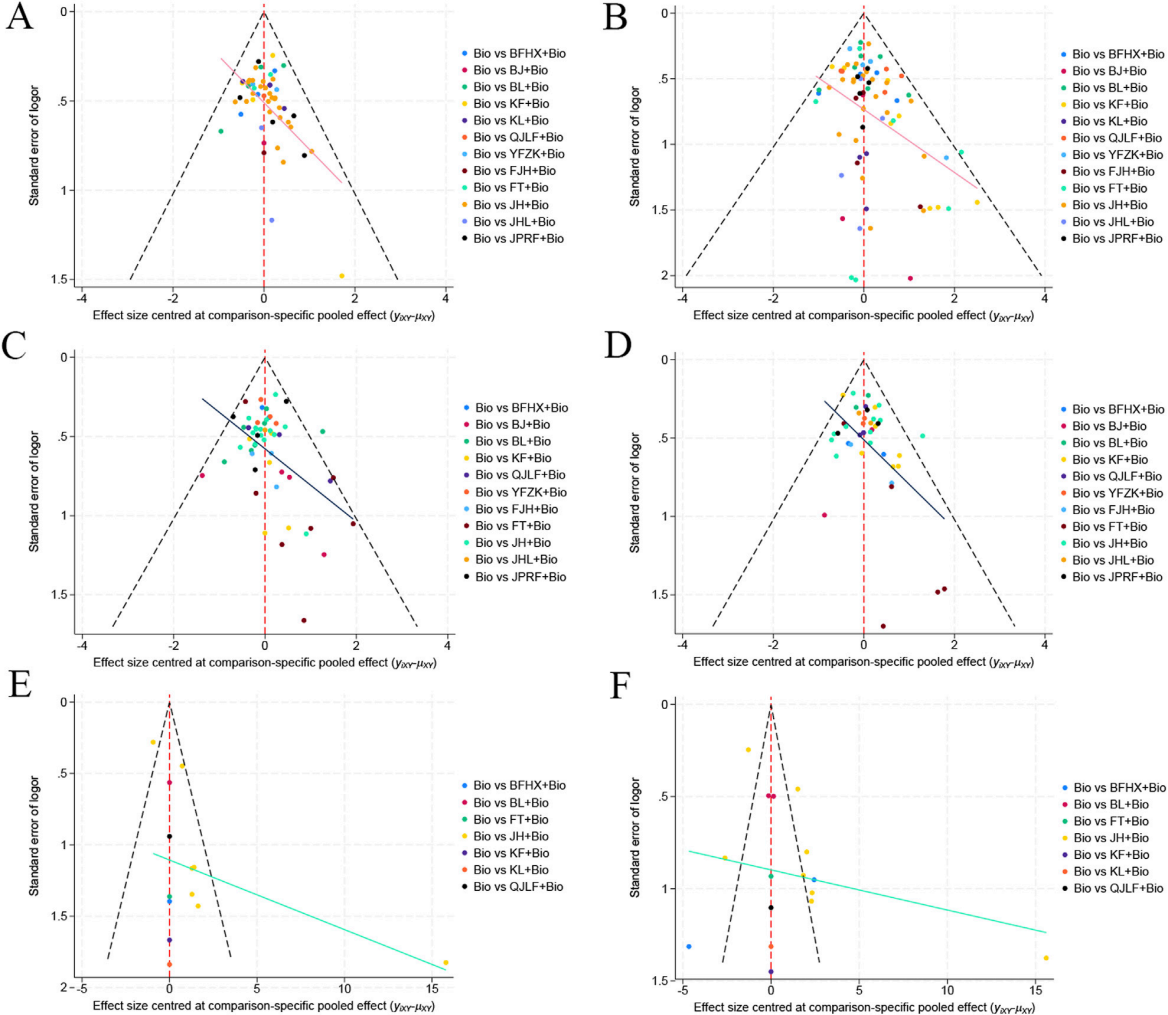
Comparison of various outcome indicators shown through adjusted funnel Plots: **(A)** Network evidence map of clinical efficacy rate; **(B)** Network evidence map of negative conversion rate; **(C)** Network evidence map of lesion absorption rate; **(D)** Network evidence map of cavity absorption rate; **(E)** Network evidence map of CD3^+^ T lymphocyte improvement rate; **(F)** Network evidence map of CD4^+^ T lymphocyte improvement rate.

This study conducted a publication bias assessment for the included literature and created a comparison-adjusted funnel plot. Due to the poor symmetry of the generated comparison-adjusted funnel plot using Egger’s test, which demonstrated statistically significant publication bias for the following outcomes: clinical efficacy (*P* = 0.001), negative conversion rate (*P* < 0.001), lesion absorption (*P* = 0.004), cavity absorption (P = 0.001), CD3^+^ T lymphocyte improvement rate (*P* = 0.049), and CD4^+^ T lymphocyte improvement rate (*P* = 0.027), as visualized in Figure 6.

## 4 Discussion

*Mycobacterium tuberculosis*, a Gram-positive *bacillus* characterized by a unique lipid-rich, utilizes this structural feature for host survival and antimicrobial resistance (Gagneux, 2012). Following phagocytosis by alveolar macrophages during the initial infection, the bacterium replicates intracellularly due to their lipid membrane composition, thereby evading immune clearance (Pieters, 2008). In the pathological process of pulmonary tuberculosis, both the immune response and the direct toxic effects of the bacteria cause damage to lung tissue. Common tissue damage includes necrosis, fibrosis, and cavity formation. Although partial parenchymal repair may occur, persistent inflammation and fibrosis may lead to irreversible lung function impairment, ultimately affecting the quality of life among patients.

TCM classifies pulmonary tuberculosis as “lung tuberculosis”, attributing its pathogenesis to dual mechanisms: deficiency of vital energy and invasion by the tuberculosis pathogen. The former refers to insufficient vital energy, while the latter refers to the invasion of the “tuberculosis insect”, which damages the lung yin, leading to lung yin deficiency, and may progressively develop into both qi and yin deficiency. Progressive disease evolution may induce depletion of yin with yang transformation, potentially culminating in severe dual yin-yang deficiency without timely intervention (Cheng-Li et al., 2021). Therefore, TCM treatment for TB emphasizes both tonifying resistance and eliminating the pathogen: tonifying the body by strengthening the immune system, including methods like replenishing qi and blood, strengthening the spleen, and boosting energy; eliminating the pathogen involves targeting the removal of TB pathogens and their associated pathological changes, often using methods like nourishing the yin, moistening the lungs, generating body fluids, and detoxifying.

This study synthesizes 100 RCTs, including 12 types of oral TCMs, and analyzes six outcome indicators. Indirect comparisons among included oral TCMs suggest optimal therapeutic outcomes with combined oral TCM and standard biomedicine. The network meta-analysis indicates that the combination of FeiJieHe Pill with standard biomedicine shows strong effects in improving clinical efficacy and cavity absorption rates. FeiJieHe Pill contain threekey medicinal metabolites: Earthworm, *Bletilla striata*, and prepared *Polygonum multiflorum*. Modern pharmacological studies indicat the capacity of Earthworm to downregulate Bcl-2-associated X protein expression while upregulating B-cell lymphoma-2 protein expression, conferring a good immune-enhancing effect (Cheng-Li et al., 2021). *Bletilla striata* polysaccharides exhibit strong adhesion properties, forming a protective membrane on wound surfaces and adjusting the t-PA/PAI-1 ratio, restoring the balance of the coagulation system (Yuxuan et al., 2018; Jia, 2022). Prepared *Polygonum multiflorum*, a traditional tonic, demonstrates validated immunoenhancing activity in modern pharmacology (Ya et al., 2019; Donglin et al., 2021). In terms of improving negative conversion rates, KangLao Pill combined with standard biomedicine exhibited strong effects. KangLao Pill is primarily composed of two botanical drugs: Siegesbeckia orientalis and Morus root bark. Modern pharmacological studies reveal that the metabolites of Siegesbeckia orientalis (Chrysophanol I and II) exhibit strong inhibitory effects on*Mycobacterium tuberculosis* (Buhan et al., 1980), while Mulberry Root Bark Polysaccharides (PMA) are a unique plant polysaccharide that can regulate spleen lymphocytes in mice and enhance immune modulation (Kim et al., 2000). In parallel, Bu Jin Tablet combined with standard biomedicine exhibited optimal efficacy for lesion absorption rate improvement. This formulation integrates traditional restorative agents: antler gelatin, silkworm, tortoise shell gelatin, red ginseng, polygonatum, Poria, and gecko, all traditional tonics. Modern pharmacological studies validate immune-enhancing or anti-aging effects of these botanical metabolites (Fang and Dao-Pei:, 2006; Xiue et al., 2014; Lili et al., 2018; Chen et al., 2020; Hongying et al., 2021; Ping et al., 2022). In terms of improving the improvement rate of CD3^+^ T lymphocytes and CD4^+^ T lymphocytes, the combination of Tuberculosis Pills with conventional biomedicine shows significant advantages. JieHe Pill incorporates three principalmetabolites: turtle shell, stem of hundred-leaf, and soft-shelled turtle shell. Modern pharmacological research has shown that turtle shell contains various active metabolites such as animal gelatin, collagen, protein, calcium, and phosphorus, which can enhance immunity through Th1 and Th2 cells modulation while exerting antibacterial and anti-inflammatory effects (Donglin et al., 2021). The stem of hundred-leaf contains multiple metabolites like stem alkaloids, proto-stem alkaloids, and deoxy-stem alkaloids, which can regulate anchor protein subtype 1 to relax smooth muscles, and have expectorant and antitussive effects (Chang et al., 2022). Soft-shelled turtle shell similarly contains animal gelatin, bone collagen, calcium, and phosphorus that can improve immunity, enhance hematopoietic function, and effectively reduce inflammation factor levels (Wenjia et al., 2023). Concerning adverse reactions, the addition of oral TCM to standard anti-TB drugs demonstrated no increased adverse reactions. On the contrary, among 60 studies reporting adverse reactions, the experimental group had fewer adverse reactions than the control group. However, the definitions and descriptions of adverse reactions varied among different studies, making quantitative evaluation difficult. Therefore, standardized reporting of adverse reactions to oral TCM is crucial for guiding clinical practice, assessing safety, and providing reliable decision-making support. Regarding publication bias, clinical efficacy, negative conversion rate, lesion absorption rate, cavity absorption rate, and CD4^+^ T lymphocyte improvement rate all showed varying degrees of publication bias, potentially compromising the reliability of these findings.

This study has several limitations that should be acknowledged. First, the overall methodological quality of the included studies was moderate. None of the studies reported allocation concealment, and only one study employed a double-blind design while another used a single-blind design. The lack of blinding and allocation concealment may introduce selection bias and detection bias, thereby reducing the credibility of the results. Second, although all included CCPPs were labeled in the original studies as having adjuvant anti-tuberculosis effects, there is a lack of direct comparisons between different CCPPs. Consequently, the comparative results in this study are derived entirely from indirect evidence and should be interpreted with caution. Moreover, for immunological outcomes such as the improvement rates of CD3^+^ and CD4^+^ T lymphocytes, some intervention combinations demonstrated statistically significant effects; however, the wide confidence intervals suggest potential small-sample effects, which may undermine the stability of the findings. Third, most included studies did not report medication adherence or loss to follow-up, which may confound the assessment of treatment efficacy. Due to certain limitations inherent in studies on commercial Chinese polyherbal preparation, not all of the original publications provided detailed information on extraction processes or chemical fingerprinting. The available information was mainly obtained from drug package inserts or the Pharmacopoeia of the *Pharmacopoeia of the People’s Republic of China* (Commission, 2020). Finally, there was inconsistency in the definition of “clinical efficacy rate” across studies, which may have introduced substantial heterogeneity in the pooled analysis and affected the robustness of the conclusions.

## 5 Conclusion

The integration of oral TCMs combined with standard biomedicine in the included studies demonstrated enhanced efficacy across at least one outcome measure. Among these regimens, FeiJieHe Pill combined with conventional biomedicine exhibits optimal efficacy for improving clinical efficacy and cavity absorption rate. JieHe Pill combined with conventional biomedicine similarly shows superior efficacy regarding the enhancement of the recovery rate of CD3^+^ T lymphocytes and CD4^+^ T lymphocytes. KangLao Pill combined with standard biomedicine achieves maximal improvement in negative conversion rates, while Bu Jin Tablet combined with standard biomedicine demonstrates the greatest effectiveness for lesion absorption rate improvement. The conclusions of this study also require verification through multi-center, large-sample, high-quality randomized controlled trials.

## Abbreviations

T, Experimental group; C, Control group; Bio, biomedicine; —, Not reported; BL, Bailing Capsule; BFHX, Bu Fei HuoXue Capsule; BJ, Bu Jin Tablet; FJH, FeiJieHe Pill; FT, Fei Tai Capsule; JPRF, Jian Pi Run Fei Pill; JHL, Jie He Ling Tablet; JHW, Jie He Pill; KF, KangFu Xin Liquid; KL, KangLao Pill; QJLFJN, QiJia LiFei Capsule; YFZKJN, YiFei ZhiKe Capsule.
