# Supplementary material for: Oral commercial Chinese polyherbal preparations combined with conventional biomedicine for pulmonary tuberculosis: network meta-analysis
Source: Front Pharmacol. 2025 Oct 28;16:1588586. doi: 10.3389/fphar.2025.1588586 (PMC12602424; doi:10.3389/fphar.2025.1588586)
Supplement: Supplementary file 1 [file Supplementaryfile1.docx]

Pubmed（1056）

Subject Headings：Tuberculosis, Pulmonary

Keywords：Pulmonary Consumption;

Consumption, Pulmonary;

Consumptions, Pulmonary;

Pulmonary Consumptions;

Pulmonary Phthisis;

Phthises, Pulmonary;

Phthisis, Pulmonary;

Pulmonary Phthises;

Pulmonary Tuberculoses;

Pulmonary Tuberculosis;

Tuberculoses, Pulmonary.

Subject Headings: Traditional Chinese Medicine

Keywords：Zhong Yi Xue

Chung I Hsueh

Hsueh, Chung I

Traditional Medicine, Chinese

Chinese Traditional Medicine

Traditional Chinese Medicine

Chinese Medicine, Traditional

Traditional Tongue Diagnosis

Tongue Diagnoses, Traditional

Tongue Diagnosis, Traditional

Traditional Tongue Diagnoses

Traditional Tongue Assessment

Tongue Assessment, Traditional

Traditional Tongue Assessments

((((((((((((Tuberculosis, Pulmonary[MeSH Terms]) OR (Pulmonary Consumption[Title/Abstract])) OR (Consumption, Pulmonary[Title/Abstract])) OR (Consumptions, Pulmonary[Title/Abstract])) OR (Pulmonary Consumptions[Title/Abstract])) OR (Pulmonary Phthisis[Title/Abstract])) OR (Phthises, Pulmonary[Title/Abstract])) OR (Phthisis, Pulmonary[Title/Abstract])) OR (Pulmonary Phthises[Title/Abstract])) OR (Pulmonary Tuberculoses[Title/Abstract])) OR (Pulmonary Tuberculosis[Title/Abstract])) OR (Tuberculoses, Pulmonary[Title/Abstract])) AND (((((((((((((((Traditional Chinese Medicine[MeSH Terms]) OR (Zhong Yi Xue[Title/Abstract])) OR (Chung I Hsueh[Title/Abstract])) OR (Hsueh, Chung I[Title/Abstract])) OR (Traditional Medicine, Chinese[Title/Abstract])) OR (Chinese Traditional Medicine[Title/Abstract])) OR (Traditional Chinese Medicine[Title/Abstract])) OR (Chinese Medicine, Traditional[Title/Abstract])) OR (Traditional Tongue Diagnosis[Title/Abstract])) OR (Tongue Diagnoses, Traditional[Title/Abstract])) OR (Tongue Diagnosis, Traditional[Title/Abstract])) OR (Traditional Tongue Diagnoses[Title/Abstract])) OR (Traditional Tongue Assessment[Title/Abstract])) OR (Tongue Assessment, Traditional[Title/Abstract])) OR (Traditional Tongue Assessments[Title/Abstract]))

Embase（283）

Subject Headings：lung tuberculosis

Keywords：'bronchial tuberculosis' OR 'bronchitis tuberculosa' OR 'bronchus tuberculosis' OR 'cavernous lung tuberculosis' OR 'cavernous pulmonary tuberculosis' OR 'cavernous tuberculosis' OR 'cavitary lung tuberculosis' OR 'cavitary tuberculosis' OR 'chronic lung tuberculosis' OR 'chronic pulmonary tuberculosis' OR 'chronic tuberculosis, lung' OR 'coniotuberculosis' OR 'lung caseation' OR 'lung TB' OR 'lung tuberculosis epidemiology' OR 'lung tuberculosis treatment' OR 'lung tuberculous cavity' OR 'phthisis' OR 'pneumonophthisis' OR 'pneumophthisiology' OR 'pulmonary TB' OR 'pulmonary tuberculosis' OR 'tiocarlide chronic lung tuberculosis' OR 'tuberculosis bronchi' OR 'tuberculosis pulmonis' OR 'tuberculosis, lung' OR 'tuberculosis, pulmonary' OR 'tuberculous bronchitis' OR 'lung tuberculosis'

Subject Headings：Chinese medicine

Keywords：'Chinese herbal medicine' OR 'Chinese traditional medicine' OR 'medicine, Chinese traditional' OR 'traditional Chinese medicine' OR 'Chinese medicine'

**#3**

#1 AND #2

[283](https://embase.pubapi.xyz/)

**#2**

**'chinese herbal medicine'**/exp OR **'chinese herbal medicine'** OR **'chinese traditional medicine'**/exp OR **'chinese traditional medicine'** OR **'medicine, chinese traditional'**/exp OR **'medicine, chinese traditional'** OR **'traditional chinese medicine'**/exp OR **'traditional chinese medicine'** OR **'chinese medicine'**/exp OR **'chinese medicine'**

[292,240](https://embase.pubapi.xyz/)

**#1**

**'bronchial tuberculosis'**/exp OR **'bronchial tuberculosis'** OR **'bronchitis tuberculosa'**/exp OR **'bronchitis tuberculosa'** OR **'bronchus tuberculosis'**/exp OR **'bronchus tuberculosis'** OR **'cavernous lung tuberculosis'**/exp OR **'cavernous lung tuberculosis'** OR **'cavernous pulmonary tuberculosis'**/exp OR **'cavernous pulmonary tuberculosis'** OR **'cavernous tuberculosis'**/exp OR **'cavernous tuberculosis'** OR **'cavitary lung tuberculosis'**/exp OR **'cavitary lung tuberculosis'** OR **'cavitary tuberculosis'**/exp OR **'cavitary tuberculosis'** OR **'chronic lung tuberculosis'**/exp OR **'chronic lung tuberculosis'** OR **'chronic pulmonary tuberculosis'**/exp OR **'chronic pulmonary tuberculosis'** OR **'chronic tuberculosis, lung'**/exp OR **'chronic tuberculosis, lung'** OR **'coniotuberculosis'**/exp OR **'coniotuberculosis'** OR **'lung caseation'**/exp OR **'lung caseation'** OR **'lung tb'**/exp OR **'lung tb'** OR **'lung tuberculosis epidemiology'**/exp OR **'lung tuberculosis epidemiology'** OR **'lung tuberculosis treatment'**/exp OR **'lung tuberculosis treatment'** OR **'lung tuberculous cavity'**/exp OR **'lung tuberculous cavity'** OR **'phthisis'**/exp OR **'phthisis'** OR **'pneumonophthisis'**/exp OR **'pneumonophthisis'** OR **'pneumophthisiology'**/exp OR **'pneumophthisiology'** OR **'pulmonary tb'**/exp OR **'pulmonary tb'** OR **'pulmonary tuberculosis'**/exp OR **'pulmonary tuberculosis'** OR **'tiocarlide chronic lung tuberculosis'**/exp OR **'tiocarlide chronic lung tuberculosis'** OR **'tuberculosis bronchi'**/exp OR **'tuberculosis bronchi'** OR **'tuberculosis pulmonis'**/exp OR **'tuberculosis pulmonis'** OR **'tuberculosis, lung'**/exp OR **'tuberculosis, lung'** OR **'tuberculosis, pulmonary'**/exp OR **'tuberculosis, pulmonary'** OR **'tuberculous bronchitis'**/exp OR **'tuberculous bronchitis'** OR **'lung tuberculosis'**/exp OR **'lung tuberculosis'**

Cochranelibrary（18）

Subject Headings：Tuberculosis, Pulmonary

Keywords： Pulmonary Phthisis;

Pulmonary Tuberculoses;

Phthises, Pulmonary;

Pulmonary Tuberculosis;

Consumption, Pulmonary;

Pulmonary Consumptions;

Consumptions, Pulmonary;

Phthisis, Pulmonary;

Tuberculoses, Pulmonary;

Pulmonary Consumption;

Pulmonary Phthises

Subject Headings：Medicine, Chinese Traditional

Keywords：Chinese Medicine, Traditional;

Hsueh, Chung I;

Traditional Chinese Medicine;

Zhong Yi Xue;

Traditional Medicine, Chinese;

Chung I Hsueh;

Chinese Traditional Medicine;

Tongue Diagnoses, Traditional;

Traditional Tongue Assessments;

Traditional Tongue Diagnoses;

Tongue Assessment, Traditional;

Traditional Tongue Assessment;

Tongue Diagnosis, Traditional;

Traditional Tongue Diagnosis.

Search Name:

Date Run: 22/01/2025 10:40:33

Comment:

ID Search Hits

#1 MeSH descriptor: [Tuberculosis, Pulmonary] explode all trees 1443

#2 (Pulmonary Phthisis):ti,ab,kw OR (Pulmonary Tuberculoses):ti,ab,kw OR (Phthises, Pulmonary):ti,ab,kw OR (Pulmonary Tuberculosis):ti,ab,kw OR (Consumption, Pulmonary):ti,ab,kw (Word variations have been searched) 6231

#3 (Pulmonary Consumptions):ti,ab,kw OR (Consumptions, Pulmonary):ti,ab,kw OR (Phthisis, Pulmonary):ti,ab,kw OR (Tuberculoses, Pulmonary):ti,ab,kw OR (Pulmonary Consumption):ti,ab,kw (Word variations have been searched) 3163

#4 (Pulmonary Phthises):ti,ab,kw (Word variations have been searched) 0

#5 #1 OR #2 OR #3 OR #4 6233

#6 MeSH descriptor: [Medicine, Chinese Traditional] explode all trees 1829

#7 (Chinese Medicine, Traditional):ti,ab,kw OR (Hsueh, Chung I):ti,ab,kw OR (Traditional Chinese Medicine):ti,ab,kw OR (Zhong Yi Xue):ti,ab,kw OR (Traditional Medicine, Chinese):ti,ab,kw (Word variations have been searched) 12079

#8 (Chung I Hsueh):ti,ab,kw OR (Chinese Traditional Medicine):ti,ab,kw OR (Tongue Diagnoses, Traditional):ti,ab,kw OR (Traditional Tongue Assessments):ti,ab,kw OR (Traditional Tongue Diagnoses):ti,ab,kw (Word variations have been searched) 12147

#9 (Tongue Assessment, Traditional):ti,ab,kw OR (Traditional Tongue Assessment):ti,ab,kw OR (Tongue Diagnosis, Traditional):ti,ab,kw (Word variations have been searched) 193

#10 #6 OR #7 OR #8 OR #9 12428

#11 #5 AND #10 18

Web of sci（460）

**(((((((((((TS=(Tuberculosis, Pulmonary)) OR TS=(Pulmonary Consumption)) OR TS=(Consumption, Pulmonary)) OR TS=(Consumptions, Pulmonary)) OR TS=(Pulmonary Consumptions)) OR TS=(Pulmonary Phthisis)) OR TS=(Phthises, Pulmonary)) OR TS=(Phthisis, Pulmonary)) OR TS=(Pulmonary Phthises)) OR TS=(Pulmonary Tuberculoses)) OR TS=(Pulmonary Tuberculosis)) OR TS=(Tuberculoses, Pulmonary)** and **Preprint Citation Index** (Exclude – Database) AND **TS=(Chinese Medicine, Traditional)) OR TS=(Traditional Tongue Diagnosis)) OR TS=(Tongue Diagnoses, Traditional)) OR TS=(Tongue Diagnosis, Traditional)) OR TS=(Traditional Tongue Diagnoses)) OR TS=(Traditional Tongue Assessment)) OR TS=(Tongue Assessment, Traditional)) OR TS=(Traditional Tongue Assessments)** and **Preprint Citation Index** (Exclude – Database)

<https://www.webofscience.com/wos/alldb/summary/e10d1e10-b2a8-4e31-ae96-40c5360d8706-0145391db8/relevance/1>

CNKI

| 检索主题：口服中成药结合西药治疗肺结核的网状meta分析 |
| --- |
| 检索范围：总库 |
| 检索年限：不限 |
| 检索时间：2025-01-22 |
| 检索式A：（主题：肺结核 + 结核病 + 结核杆菌 + 结核性肺病 + 肺部结核 + 肺痨 + 痨病）AND（主题：中药 + 中成药 + 中西医 + 中西医结合 + 丸 + 丹 + 散 + 片 + 口服液 + 颗粒 + 剂 + 胶囊 + 汤）AND（篇关摘：随机 + 随机对照 + 随机研究 + 临床实验 + 临床研究 + 安慰剂(模糊)） |

VIP

[((((((((任意字段=肺结核 OR 任意字段=结核病) OR 任意字段=结核杆菌) OR 任意字段=结核性肺病) OR 任意字段=肺部结核) OR 任意字段=肺痨) OR 任意字段=痨病) AND ((((((((((((任意字段=中药 OR 任意字段=中成药) OR 任意字段=中西医) OR 任意字段=中西医结合) OR 任意字段=丸) OR 任意字段=丹) OR 任意字段=散) OR 任意字段=片) OR 任意字段=口服液) OR 任意字段=颗粒) OR 任意字段=剂) OR 任意字段=胶囊) OR 任意字段=汤)) AND (((((任意字段=随机 OR 任意字段=随机对照) OR 任意字段=随机研究) OR 任意字段=临床实验) OR 任意字段=临床研究) OR 任意字段=安慰剂))](http://qikan.cqvip.com/Qikan/search/index?LngMySearHistoryIdGuid=2c42a8dd-d0e5-473c-8db4-06c14d63c18e&from=Qikan_Article_History)

WanFang

[主题:(肺结核 OR 结核病 OR 结核杆菌 OR 结核性肺病 OR 肺部结核 OR 肺痨 OR 痨病) and 主题:(中药 OR 中成药 OR 中西医 OR 中西医结合 OR 丸 OR 丹 OR 散 OR 片 OR 口服液 OR 颗粒 OR 剂 OR 胶囊 OR 汤) and 主题:(随机 OR 随机对照 OR 随机研究 OR 临床实验 OR 临床研究 OR 安慰剂)](https://s.wanfangdata.com.cn/advanced-search/paper?q=%E4%B8%BB%E9%A2%98%3A(%E8%82%BA%E7%BB%93%E6%A0%B8%20OR%20%E7%BB%93%E6%A0%B8%E7%97%85%20OR%20%E7%BB%93%E6%A0%B8%E6%9D%86%E8%8F%8C%20OR%20%E7%BB%93%E6%A0%B8%E6%80%A7%E8%82%BA%E7%97%85%20OR%20%E8%82%BA%E9%83%A8%E7%BB%93%E6%A0%B8%20OR%20%E8%82%BA%E7%97%A8%20OR%20%E7%97%A8%E7%97%85)%20and%20%E4%B8%BB%E9%A2%98%3A(%E4%B8%AD%E8%8D%AF%20OR%20%E4%B8%AD%E6%88%90%E8%8D%AF%20OR%20%E4%B8%AD%E8%A5%BF%E5%8C%BB%20OR%20%E4%B8%AD%E8%A5%BF%E5%8C%BB%E7%BB%93%E5%90%88%20OR%20%E4%B8%B8%20OR%20%E4%B8%B9%20OR%20%E6%95%A3%20OR%20%E7%89%87%20OR%20%E5%8F%A3%E6%9C%8D%E6%B6%B2%20OR%20%E9%A2%97%E7%B2%92%20OR%20%E5%89%82%20OR%20%E8%83%B6%E5%9B%8A%20OR%20%E6%B1%A4)%20and%20%E4%B8%BB%E9%A2%98%3A(%E9%9A%8F%E6%9C%BA%20OR%20%E9%9A%8F%E6%9C%BA%E5%AF%B9%E7%85%A7%20OR%20%E9%9A%8F%E6%9C%BA%E7%A0%94%E7%A9%B6%20OR%20%E4%B8%B4%E5%BA%8A%E5%AE%9E%E9%AA%8C%20OR%20%E4%B8%B4%E5%BA%8A%E7%A0%94%E7%A9%B6%20OR%20%E5%AE%89%E6%85%B0%E5%89%82)&searchtype=expert&type=%5b%22periodical%22%5d)

sinomed

[("随机"[常用字段:智能] OR "随机对照"[常用字段:智能] OR "随机研究"[常用字段:智能] OR "临床实验"[常用字段:智能] OR "临床研究"[常用字段:智能] OR "安慰剂"[常用字段:智能]) AND (("颗粒"[常用字段:智能] OR "剂"[常用字段:智能] OR "胶囊"[常用字段:智能] OR "汤"[常用字段:智能]) OR ("中药"[常用字段:智能] OR "中成药"[常用字段:智能] OR "中西医"[常用字段:智能] OR "中西医结合"[常用字段:智能] OR "丸"[常用字段:智能] OR "丹"[常用字段:智能] OR "散"[常用字段:智能] OR "片"[常用字段:智能] OR "口服液"[常用字段:智能])) AND ("肺结核"[常用字段:智能] OR "结核病"[常用字段:智能] OR "结核杆菌"[常用字段:智能] OR "结核性肺病"[常用字段:智能] OR "肺部结核"[常用字段:智能] OR "肺痨"[常用字段:智能] OR "痨病"[常用字段:智能])](javascript:toDoRelimitSearch();)
