# Supplementary material for: Oral commercial Chinese polyherbal preparations combined with conventional biomedicine for pulmonary tuberculosis: network meta-analysis
Source: Front Pharmacol. 2025 Oct 28;16:1588586. doi: 10.3389/fphar.2025.1588586 (PMC12602424; doi:10.3389/fphar.2025.1588586)
Supplement: Supplementary file 2 [file Supplementaryfile3.docx]

| Drug name | Adverse drug reactions | Contraindication or Precautions | Pharmacological Effects | Approval Number | Implementation Standard |
| --- | --- | --- | --- | --- | --- |
| Bai Ling Capsule (BL) | Not yet clear | Not yet clear | This product can reduce serum creatinine, blood urea nitrogen (BUN), urinary protein content, and histopathological scores in rats with nephrectomy and gentamicin-induced renal injury. | Z10910036 | Pharmacopoeia of the People's Republic of China (2020 Edition, Part I) |
| Bu Fei Huo Xue Capsule（BFHX） | Monitoring data show the following adverse reactions may occur with this product:  Gastrointestinal system: Nausea, vomiting, abdominal pain, diarrhea, constipation, gastric discomfort, dry mouth, etc.  Hepatobiliary system: Abnormal liver function. Severe cases of liver injury and hepatic failure have been reported.  Skin and appendages: Rash, pruritus, urticaria.  Cardiovascular system: Palpitations.  Others: Dizziness, headache, head fullness, insomnia, etc. | This product is contraindicated in: (1) individuals allergic to its components; (2) patients with history of drug-induced liver injury, autoimmune liver disease, cirrhosis, or liver cancer; (3) cases showing abnormal liver biochemical markers suggesting hepatotoxicity. | Pharmacological studies have demonstrated that this product can reduce red blood cell (RBC) count, white blood cell (WBC) count, platelet count, and hemoglobin levels in FeCl₃-induced cor pulmonale rabbit models. It decreases whole blood viscosity, plasma viscosity, and hematocrit, while moderately reducing the abnormal rate of pulmonary P waves. Additionally, it lowers the partial pressure of carbon dioxide (PaCO₂) in blood gas and improves oxygen partial pressure (PaO₂) and oxygen saturation.  The product promotes the generation of serum hemolysin and splenic hemolytic plaque-forming cells in mice. In anesthetized dogs, it increases cardiac output and coronary blood flow, while reducing total peripheral vascular resistance, coronary resistance, myocardial oxygen consumption, and oxygen consumption index.  Furthermore, it prolongs the cough latency period and reduces coughing frequency in ammonia water-induced cough models in mice. It also extends the asthma latency period in histamine-induced asthmatic guinea pigs. | Z20030063 | Pharmacopoeia of the People's Republic of China (2020 Edition, Part I) |
| Bu Jin Tablet(BJ) | Not yet clear | Not yet clear | Not applicable (N/A) | Z22023500 | YBZ04092004 |
| FeiJieHe Pill(FJH) | Not yet clear | Not yet clear | Not applicable (N/A) | Z34020948 | WS3-B-1799-94 |
| Fei Tai Capsule(FT) | Not yet clear | Not yet clear | This product prolonged the mean survival days in mice infected with Mycobacterium tuberculosis H37Rv; mitigated weight loss in M. tuberculosis-infected guinea pigs, reduced spleen/lung weights and pathological indices, and inhibited bacterial growth in the spleen, lungs, liver, and lymph nodes; extended the cough latency period in mice induced by sulfur dioxide and ammonia inhalation, and exhibited mild hemostatic effects in tail-tip bleeding models. | Z20133021 | YBZ12862004-2012（Z） |
| Jian Pi Run Fei Pill(JPRF) | Not yet clear | Not yet clear | Not applicable (N/A) | Z20025862 | WS-10624(ZD-0624)-2002-2012Z |
| Jie He Ling Tablet（JHL） | Not yet clear | Contraindicated during pregnancy | Not applicable (N/A) | Z20063915 | YBZ12532006 |
| Jie He Pill（JH） | Not yet clear | Not yet clear | Not applicable (N/A) | Z200225187 | WS-10172-(ZD-0172)-2002 |
| Kangfuxin Liquid(KF) | Monitoring data indicate the following adverse reactions may occur with this product:  1.Gastrointestinal system: Nausea, vomiting, abdominal distension, abdominal pain, diarrhea.  2.Application site: Redness, pain.  3.Skin: Rash, pruritus.  4.Others: Dizziness, headache, chest tightness, palpitations, flushing, dyspnea, allergic reactions; Individual case reports of local numbness and anaphylactic shock. | 1.Hypersensitivity to this product or any of its components  2.Patients with asthma  3.Pregnancy | Not applicable (N/A) | Z43020995 | WS3-B-3674-2000(Z) |
| KangLao Pill（KL） | Not yet clear | Not yet clear | Not applicable (N/A) | Z44020460 | WS3-B-1336-93 |
| Qi Jia Li Fei Capsule（QJLF） | When used in combination with anti-tuberculosis chemotherapeutic agents, elevated transaminases and other adverse reactions attributed to the anti-TB drugs have been occasionally observed. These abnormalities typically resolve with symptomatic treatment. | Avoid consumption of:  1.Raw and cold foods (e.g. uncooked vegetables, chilled beverages)  2.Spicy irritants (chili peppers, pungent seasonings)  3.Animal-derived fatty foods (pork lard, tallow, fatty meats) | Pharmacological studies demonstrate that:  1.Antitussive effects: Prolongs cough latency in sulfur dioxide-induced cough models in mice  2.Hemostatic effects: Shortens coagulation time in murine models  3.Anti-inflammatory effects: Inhibits xylene-induced ear edema in mice and cotton pellet granuloma formation in rats  4.Immunomodulatory effects:  Increases thymus weight and hemolysin levels in cyclophosphamide-induced immunocompromised mice  Enhances phagocytic function of peritoneal macrophages against chicken erythrocytes in prednisone acetate-induced immunosuppressed mice  5.Antimycobacterial effects: Reduces Mycobacterium tuberculosis load in spleens of infected guinea pigs | Z20050779 | YBZ28352005-2013Z |
| Yi Fei Zhi Ke Capsule（YFZK） | Not yet clear | 1.Pregnant women  2.Women during menstruation | Not applicable (N/A) | Z20026362 | WS-10913(ZD-0913)-2002-2012Z |
