# Supplementary material for: Oral commercial Chinese polyherbal preparations combined with conventional biomedicine for pulmonary tuberculosis: network meta-analysis
Source: Front Pharmacol. 2025 Oct 28;16:1588586. doi: 10.3389/fphar.2025.1588586 (PMC12602424; doi:10.3389/fphar.2025.1588586)
Supplement: Supplementary file 3 [file Supplementaryfile2.docx]

| **Drug name** | **Botanical drug name** | **indication** |
| --- | --- | --- |
| Bai Ling Capsule (BL) | *Hirsutella sinensis* Liu, Guo, Yu et Zeng (1989) [Ophiocordycipitaceae*, Hirsutellae Mycelium*] | Tonifies the lungs and kidneys, and nourishes essence and qi.Indicated for cough, asthma, hemoptysis (coughing up blood), soreness of the lower back and legs, facial puffiness, and frequent, clear urination at night caused by deficiency of both lung and kidney.Also used as an adjuvant treatment for chronic bronchitis and chronic renal insufficiency. |
| Bu Fei Huo Xue Capsule（BFHX） | *Astragalus membranaceus* (Fisch.) Bge*.*[Fabaceae*, Astragali Radix*],  *Paeonia lactiflora* Pall*.*[Ranunculaceae*, Paeoniae Radix*]  *Psoralea corylifolia* L. [Fabaceae, *Psoraleae Fructus*] | Tonifies qi and activates blood circulation, nourishes the lungs and strengthens the kidneys.  Indicated for pulmonary heart disease (in remission stage) of the pattern of qi deficiency and blood stasis, with symptoms such as cough and shortness of breath, or wheezing with chest tightness, palpitations and dyspnea, cold limbs and fatigue, soreness and weakness of the lower back and knees, cyanosis of the lips, pale tongue with white coating, or dark purplish tongue. |
| Bu Jin Tablet(BJ) | *Citrus reticulata* Blanco [Rutaceae, *Citri Reticulatae Pericarpium*]  *Rana temporaria chensinensis* David [Ranidae, *Ranae Oviductus*]  *Cervi Cornu* [Cervidae*, Cervi Colla Cornus*]  *Zaocys dhumnades* (Cantor) [Colubridae, *Zaocys Corpus*]  *Placenta Hominis* [Hominidae, *Placentae Hominis Colla*]  *Oleum Ovi Gallinacei* [Gallus gallus domesticus, *Oleum Ovi Gallinacei Colla*]  *Platycodon grandiflorus* (Jacq.) A. DC. [Campanulaceae, *Platycodi Radix*]  *Chinemys reevesii* (Gray) [Emydidae, *Testudinis Carapax Colla*]  *Stemona sessilifolia* (Miq.) Miq.[Stemonaceae, *Stemonae Radix*]  *Fritillaria thunbergii* Miq. [Liliaceae, *Fritillariae Thunbergii Bulbus*]  *Panax ginseng* C.A. Meyer [Araliaceae, *Ginseng Radix Rubra*]  *Bletilla striata* (Thunb.) Rchb. f. [Orchidaceae, *Bletillae Rhizoma*]  *Polygonatum sibiricum* Redouté [Liliaceae, *Polygonati Rhizoma*]  *Poria cocos* (Schw.) Wolf [Polyporaceae, *Poria*]  *Gecko gecko* Linnaeus [Gekkonidae, *Gecko Corpus*]  *Ophiopogon japonicus* (L.f) Ker-Gawl. [Liliaceae, *Ophiopogonis Radix*]  *Juglans regia* L. [Juglandaceae, *Juglandis Semen*]  *Angelica sinensis* (Oliv.) Diels [Apiaceae, *Angelicae Sinensis Radix*] | Tonifies the kidneys and benefits the lungs, strengthens the spleen and resolves phlegm, relieves cough and calms asthma.Indicated for pulmonary tuberculosis, chronic bronchitis, pulmonary emphysema, and pulmonary heart disease in the remission stage. |
| FeiJieHe Pill(FJH) | *Polygonum multiflorum* Thunb. [Polygonaceae, *Polygoni Multiflori Radix Praeparata*]  *Bletilla striata* (Thunb.) Rchb. f. [Orchidaceae, *Bletillae Rhizoma*]  *Eupolyphaga sinensis* Walker [Corydiidae, *Eupolyphaga Corpus*] | Astringes yin and tonifies the lungs.  Indicated for pulmonary cavities and pulmonary hemorrhage. |
| Fei Tai Capsule(FT) | *Ixeris dentata* (Thunb.) Nakai [Asteraceae, *Ixeridis Herba*]  *Scutellaria baicalensis* Georgi [Lamiaceae, *Scutellariae Radix*]  *Glehnia littoralis* F. Schmidt ex Miq. [Apiaceae, *Glehniae Radix*]  *Trichosanthes kirilowii* Maxim. [Cucurbitaceae, *Trichosanthis Fructus*]  *Pseudostellaria heterophylla* (Miq.) Pax ex Pax & Hoffm. [Caryophyllaceae, *Pseudostellariae Radix*]  *Stemona sessilifolia* (Miq.) Miq. [Stemonaceae, *Stemonae Radix*]  *Eriobotrya japonica* (Thunb.) Lindl. [Rosaceae, *Eriobotryae Folium*]  *Fritillaria cirrhosa* D. Don [Liliaceae, *Fritillariae Cirrhosae Bulbus*]  *Bletilla striata* (Thunb.) Rchb. f. [Orchidaceae, *Bletillae Rhizoma*] | Clears heat and resolves phlegm, moistens the lungs and eliminates parasites.  Used in combination with anti-tuberculosis chemotherapy for infiltrative pulmonary tuberculosis of the phlegm-heat with yin deficiency pattern.  Symptoms include: fever, hemoptysis, cough (with or without blood-streaked sputum), fatigue, poor appetite, malar flush, and night sweats.  It helps accelerate lesion absorption and relieve symptoms. |
| Jian Pi Run Fei Pill(JPRF) | *Dioscorea opposita* Thunb. [Dioscoreaceae, *Dioscoreae Rhizoma*]  *Rehmannia glutinosa* Libosch. [Scrophulariaceae, *Rehmanniae Radix*]  *Asparagus cochinchinensis* (Lour.) Merr. [Asparagaceae, *Asparagi Radix*]  *Ophiopogon japonicus* (L. f.) Ker Gawl. [Asparagaceae, *Ophiopogonis Radix*]  *Polygonatum kingianum* Coll. et Hemsl. [Asparagaceae, *Polygonati Rhizoma*]  *Polygonum multiflorum* Thunb. [Polygonaceae, *Polygoni Multiflori Radix Praeparata*]  *Astragalus membranaceus* (Fisch.) Bunge var. mongholicus (Bunge) P. K. Hsiao[Fabaceae, *Astragali Radix*]  *Poria cocos* (Schw.) Wolf [Polyporaceae, *Poria*]  *Atractylodes macrocephala* Koidz. [Asteraceae, *Atractylodis Macrocephalae Rhizoma*]  *Fritillaria cirrhosa* D. Don [Liliaceae, *Fritillariae Cirrhosae Bulbus*]  *Glehnia littoralis* Fr. Schmidt ex Miq. [Apiaceae, *Glehniae Radix*]  *Codonopsis pilosula* (Franch.) Nannf. [Campanulaceae, *Codonopsis Radix*]  *Cornus officinalis* Siebold & Zucc. [Cornaceae, *Corni Fructus*]  *Schisandra chinensis* (Turcz.) Baill. [Schisandraceae, *Schisandrae Fructus*]  *Salvia miltiorrhiza* Bunge [Lamiaceae, *Salviae Miltiorrhizae Radix et Rhizoma*]  *Gallus gallus* domesticus Brisson [Phasianidae, *Endothelium Corneum Gigeriae Galli*]  *Crataegus pinnatifida* Bunge[Rosaceae, *Crataegi Fructus*]  *Equus asinus* L. [Equidae, *Colla Corii Asini*]  *Trichosanthes kirilowii* Maxim. [Cucurbitaceae, *Trichosanthis Fructus*]  *Bletilla striata* (Thunb.) Rchb. f. [Orchidaceae, *Bletillae Rhizoma*]  *Angelica sinensis* (Oliv.) Diels [Apiaceae, *Angelicae Sinensis Radix*]  *Paeonia lactiflora* Pall. [Ranunculaceae, *Paeoniae Radix Alba*]  *Glycyrrhiza uralensis* Fisch. [Fabaceae, *Glycyrrhizae Radix et Rhizoma*]  *Lilium brownii* F. E. Brown ex Miellez[Liliaceae, *Lilii Bulbus*]  *Anemarrhena asphodeloides* Bunge [Liliaceae, *Anemarrhenae Rhizoma*]  *Bupleurum chinense* DC. [Apiaceae, *Bupleuri Radix*]  *Scutellaria baicalensis* Georgi [Lamiaceae, *Scutellariae Radix*]  *Citrus reticulata* Blanco [Rutaceae, *Citri Reticulatae Pericarpium*] | Nourishes yin and moistens the lungs, relieves cough and resolves phlegm, strengthens the spleen and stimulates the appetite.  Indicated for pulmonary tuberculosis with lung yin deficiency, presenting symptoms such as tidal fever, night sweats, cough with blood-streaked sputum, loss of appetite, shortness of breath, fatigue, and muscle wasting.  It can also be used as an adjunctive treatment for liver function impairment caused by anti-tuberculosis medications. |
| Jie He Ling Tablet（JHL） | *Euphorbia fischeriana* Steud. [Euphorbiaceae, *Euphorbiae Fischerianae Radix*] | Antitubercular.  Primarily indicated for lymph node tuberculosis, and also effective for pulmonary tuberculosis and other forms of lymph node tuberculosis. |
| Jie He Pill（JH） | *Chinemys reevesii* (Gray) [Testudinidae, *Testudinis Carapax et Plastrum*]  *Stemona tuberosa* Lour. [Stemonaceae, *Stemonae Radix*]  *Trionyx sinensis* Wiegmann [Trionychidae, *Trionycis Carapax*]  *Fluorite* [*Fluoritum*]  *Rehmannia glutinosa* (Gaertn.) Libosch. ex Fisch. & C.A. Mey. [Scrophulariaceae, *Rehmanniae Radix*]  *Rehmannia glutinosa* (Gaertn.) Libosch. ex Fisch. & C.A. Mey. [Scrophulariaceae, *Rehmanniae Radix Praeparata*]  *Asparagus cochinchinensis* (Lour.) Merr. [Liliaceae, *Asparagi Radix*]  *Glehnia littoralis* F. Schmidt ex Miq. [Apiaceae, *Glehniae Radix*]  *Ostrea gigas* Thunberg [Ostreidae, *Ostreae Concha*]  *Equus asinus* L. [Equidae, *Asini Corii Colla*]  Elephas maximus L. OR Rhinoceros unicornis L. OR Hipparion spp[*Os Draconis*]  *Ophiopogon japonicus* (L. f.) Ker-Gawl. [Liliaceae, *Ophiopogonis Radix*]  *Apis cerana* Fabricius [Apidae, *Cera Flava*]  *Rheum palmatum* L. [Polygonaceae, *Rhei Radix et Rhizoma Praeparata*]  *Bletilla striata* (Thunb.) Rchb. f. [Orchidaceae, *Bletillae Rhizoma*]  *Fritillaria cirrhosa* D. Don [Liliaceae, *Fritillariae Cirrhosae Bulbus*] | Nourishes yin and blood, tonifies the lungs and clears heat, promotes calcification of pulmonary cavities.  Used as an adjunctive treatment for pulmonary tuberculosis and bone tuberculosis. |
| Kangfuxin Liquid(KF) | *Periplaneta americana* (Linnaeus) [Blattidae, *Periplanetae Extractum*] | Indicated for blood stasis with obstruction, gastric pain with bleeding, and the treatment of gastric and duodenal ulcers;  also used as an adjunctive treatment for yin deficiency with pulmonary tuberculosis. |
| KangLao Pill（KL） | *Ardisia japonica* (Thunb.) Blume [Myrsinaceae, *Ardisiae Japonicae Herba*]  *Stemona sessilifolia* (Miq.) Miq. [Stemonaceae, *Stemonae Radix*]  *Morus alba* L. [Moraceae, *Mori Cortex*]  *Cudrania tricuspidata* (Carrière) Bureau ex Lavallée [Moraceae, *Cudraniae Radix*]  *Ficus hirta* Vahl [Moraceae, *Fici Hirtae Radix*]  *Bletilla striata* (Thunb.) Rchb. f. [Orchidaceae, *Bletillae Rhizoma*] | Invigorates blood and stops bleeding, dispels stasis and promotes tissue regeneration, resolves phlegm and relieves cough.  Indicated for infiltrative pulmonary tuberculosis with blood-streaked sputum. |
| Qi Jia Li Fei Capsule（QJLF） | *Astragalus membranaceus* (Fisch.) Bunge [Fabaceae, *Astragali Radix*]  *Trionyx sinensis* Wiegmann [Trionychidae, *Trionycis Carapax*]  *Lycium chinense* Mill. [Solanaceae, *Lycii Cortex*]  *Gekko gecko* Linnaeus [Gekkonidae, *Gecko*]  *Cordyceps sinensis* (Berk.) Sacc. [Clavicipitaceae, *Cordyceps*]  *Bletilla striata* (Thunb.) Rchb. f. [Orchidaceae, *Bletillae Rhizoma*]  *Pinctada martensii* (Dunker) [Pteriidae, *Margarita*]  *Forsythia suspensa* (Thunb.) Vahl [Oleaceae, *Forsythiae Fructus*]  *Prunella vulgaris* L. [Lamiaceae, *Prunellae Spica*]  *Zaocys dhumnades* (Cantor) [Colubridae, *Zaocys*]  *Stemona tuberosa* Lour. [Stemonaceae, *Stemonae Radix*]  *Fritillaria cirrhosa* D. Don [Liliaceae, *Fritillariae Cirrhosae Bulbus*]  *Scutellaria baicalensis* Georgi [Lamiaceae, *Scutellariae Radix*]  *Houttuynia cordata* Thunb. [Saururaceae, *Houttuyniae Herba*] | Tonifies qi and nourishes yin, reduces fever and combats tuberculosis.  Used in combination with standard anti-tuberculosis chemotherapy as an adjunctive treatment for pulmonary tuberculosis.  It can help alleviate symptoms such as fever, fatigue, shortness of breath, scanty and sticky sputum (possibly with blood or mild hemoptysis), severe cough with chest pain, afternoon tidal fever, night sweats, nausea, vomiting, and poor appetite.  It may also promote sputum (tubercle bacilli) conversion to negative and facilitate the absorption of pulmonary lesions. |
| Yi Fei Zhi Ke Capsule（YFZK） | *Ranunculus ternatus* Thunb. [Ranunculaceae, *Ranunculi Ternati Radix*]  *Panax notoginseng* (Burkill) F.H. Chen [Araliaceae, *Panacis Notoginseng Radix*]  *Bletilla striata* (Thunb.) Rchb. f. [Orchidaceae, *Bletillae Rhizoma*]  *Lilium brownii* F.E. Brown ex Miellez [Liliaceae, *Lilii Bulbus*]  *Gekko gecko* Linnaeus [Gekkonidae, *Gekko*]  *Lysionotus pauciflorus* Maxim. [Gesneriaceae, *Lysionoti Herba*]  *Stemona tuberosa* Lour. [Stemonaceae, *Stemonae Radix*] | Nourishes yin and moistens the lungs, relieves cough and eliminates phlegm.  Indicated for cough with sputum caused by chronic bronchitis. |
